# Hybrid Cements: Mechanical Properties, Microstructure and Radiological Behavior

**DOI:** 10.3390/molecules27020498

**Published:** 2022-01-13

**Authors:** Ana María Moreno de los Reyes, José Antonio Suárez-Navarro, María del Mar Alonso, Catalina Gascó, Isabel Sobrados, Francisca Puertas

**Affiliations:** 1 Department of Materials, Eduardo Torroja Institute for Construction Sciences (IETcc-CSIC), 28033 Madrid, Spain; ana.moreno@ietcc.csic.es (A.M.M.d.l.R.); mmalonso@ietcc.csic.es (M.d.M.A.); 2 Department of Environment, Environmental Radioactivity and Radiological Surveillance (CIEMAT), Avenida Complutense 40, 28040 Madrid, Spain; ja.suarez@ciemat.es (J.A.S.-N.); ctlngasleon@outlook.es (C.G.); 3 Department of Energy, Environment and Health, Institute of Material Sciences of Madrid (ICMM.CSIC), 28049 Madrid, Spain; isobrado@icmm.csic.es

**Keywords:** NORMs, hybrid cements, alkaline activation, fly ash, characterization, gamma spectrometry and radon

## Abstract

The use of more eco-efficient cements in concretes is one of the keys to ensuring construction industry sustainability. Such eco-efficient binders often contain large but variable proportions of industrial waste or by-products in their composition, many of which may be naturally occurring radioactive materials (NORMs). This study explored the application of a new gamma spectrometric method for measuring radionuclide activity in hybrid alkali-activated cements from solid 5 cm cubic specimens rather than powder samples. The research involved assessing the effect of significant variables such as the nature of the alkaline activator, reaction time and curing conditions to relate the microstructures identified to the radiological behavior observed. The findings showed that varying the inputs generated pastes with similar reaction products (C-S-H, C-A-S-H and (N,C)-A-S-H) but different microstructures. The new gamma spectrometric method for measuring radioactivity in solid 5 cm cubic specimens in alkaline pastes was found to be valid. The variables involved in hybrid cement activation were shown to have no impact on specimen radioactive content. The powder samples, however, emanated ^222^Rn (a descendent of ^226^Ra), possibly due to the deformation taking place in fly ash structure during alkaline activation. Further research would be required to explain that finding.

## 1. Introduction

Portland cement concrete is the construction material most widely used the world over. Its large output and consumption are primarily attributable to the ready availability of the raw materials nearly anywhere on the planet, the ease and diversity of its preparation [1] and its high performance in terms of both mechanical strength and durability [2,3]. Global production of concrete presently comes to over 3 Gt year^−1^. In light of expected world population growth [4], however, output is predicted to rise substantially to respond to infrastructure, housing and other building needs.

The main binder in concrete is Portland cement (OPC), which accounts for 10% to 15 wt% of the material. Cement manufacture has adverse energy and environmental implications [3,5], including high thermal and electric energy consumption rates to produce clinker and cement, the use of vast amounts of natural resources and the emission of greenhouse gases such as CO_2_. It takes 1.7 t of raw materials to manufacture 1 t of cement and the process generates 0.8 t of CO_2_ [6]. The cement industry alone is estimated to account of around 8% of planet-wide anthropogenic CO_2_.

Attempts have been underway for decades to lower the energy consumption involved in OPC manufacture and its associated environmental impact. One of the most widespread strategies is the use of supplementary cementitious materials (SCMs) to wholly or partially replace Portland cement in concrete mixes [7]. Many such SCMs, including fly ash, silica fume and blast furnace slag, are waste or by-products from other industries and many are normally occurring radioactive materials (NORMs), i.e., materials with a high radioactive content of natural origin.

The use of such NORMs in OPC manufacture is a technological necessity. It is no less necessary, however, to ensure that their inclusion in cements and concretes is radiologically safe for human health and the environment. A European Directive (2013/59/EURATOM) establishes a screening tool, the activity concentration index (ACI), to measure the impact of gamma radiation on human health. The ACI value is defined to ensure that the effective dose of external gamma radiation is not over 1 mSv a^−1^ in a standard room measuring 2.8 × 4 × 5 m^3^ enclosed by walls 20 cm thick with a density of 2.35 g m^−3^ [8]. The excess effective dose does not include the absorbed dose due to the background radiation from natural sources, defined to be 50 nGy h^−1^ [9,10]. ACI is determined for the end building material, not its individual components, from Equation (1) below:

(1) $$\mathrm{ACI}=\frac{C_{226_{Ra}}}{300}+\frac{C_{232_{Th}}}{200}+\frac{C_{40_{K}}}{3000}$$

where $C_{226_{Ra}}$, $C_{232_{Th}}$ and $C_{40_{K}}$ are, respectively, ^226^Ra, ^232^Th and ^40^K activity concentration in Bq kg^−1^ in the building material.

The ^226^Ra, ^232^Th and ^40^K activity concentrations in building materials, with or without industrial waste/by-products, are normally determined with gamma-ray spectrometry on hydrated or anhydrous powder samples. In earlier research, the authors developed a new method to determine ACI with the accuracy and precision required on solid samples (5 cm cubic specimens) of hardened Portland cement paste [11].

In that study [12] 5 cm cubic specimens were prepared with a known gamma activity concentration in which the radionuclides did not form part of the cement matrix. The findings showed that (i) the experimental values found for the powder and the 5 cm cubic samples were statistically comparable; and (ii) no differences were observed in the activity indices recorded for the various sides of the cubic specimens or at different curing times. The same methodology was subsequently applied to cement pastes with a variable type F fly ash (FA) [13] content. According to the results, ^226^Ra, ^232^Th (^212^Pb) and ^40^K activity concentrations in 28 days, 5 cm cubic specimens were statistically equal to the values for the same samples ground to a powder as well as to the theoretical activity concentrations calculated separately for the two anhydrous components. The findings also showed that Portland cement pastes with up to 30% fly ash are radiologically safe.

The present study applied the new radionuclide activity concentration measuring procedure to another type of eco-efficient cements, namely alkali-activated and in this case specifically hybrid, cements, where NORMs may account for up to 70 wt% of the total. Hybrid cements are alkali-activated materials resulting from mixing OPC with an aluminosilicate, preferably type F coal fly ash, with alkaline activators [14,15,16,17,18] such as NaOH, Na_2_CO_3_ or Na_2_SiO_3_ (waterglass), among others [19,20,21]. The reaction products primarily forming in the alkaline activation of hybrid cements include N-A-S-H and C-S-H/C-(A)-S-H -like gels, which may precipitate jointly [15,16,22,23,24,25,26,27,28,29]. Hybrid cementitious materials are characterised by the development of high early-age mechanical strength, low heat of hydration and high resistance to chemical attack, freeze-thaw cycles and fire [30,31,32,33,34,35].

Very few studies have been conducted on alkaline-activated cement and concretes (geopolymers) radiological behaviour [14,36,37,38], but none whatsoever on the hybrid cement pastes and the relationship with their microstructure. Deploying a new method developed by the authors that uses solid 5 cm cubic specimens, the present research confirmed the existence of such a relationship in hybrid (70 wt% OPC + 30 wt% FA) cement pastes. That correlation was established in different scenarios, varying the parameters involved in preparing the hybrid alkaline pastes, including the nature of the activator (either 8 M NaOH or 85% 8 M NaOH + 15% waterglass (WG)); reaction time (2 d or 28 d) and curing conditions (at 21 ± 2 °C and 99% RH, with or without thermal pre-curing at 80 °C for 20 h).

## 2. Materials and Methods

### 2.1. Materials

The hybrid cements used in this study were prepared with a CEM I 52.5 R (OPC) and a coal fly ash (FA) classified as type F in ASTM C618. The chemical compositions for the OPC and FA are given in Table 1. They were determined on a Bruker (Billerica, MA, USA) S8 Tiger XRF analyser. The loss on ignition (LoI) and insoluble residue (I.R.) values were found as specified in European standard EN 196-2:2014 [39].

The soluble silica and alumina values were established by selective attack with 1% HF [40]. The percentage of reactive silica was determined further to European standard EN 80-225:2012 [41], the 69.4% vitreous phase in FA as described in [40].

The qualitative and quantitative mineral compositions of the raw materials and pastes were recorded on a Bruker (Billerica, MA, USA) Advance AXL D8 diffractometer featuring: a Lynxeye ultrafast X-ray detector; a 2.2 kW copper anode configured without a monochromator; a high voltage generator operating at 40 kV and 20 mA; an automatic divergence slit; and a likewise automatic sample changer. The mineralogical phases detected were quantified with Rietveld analysis using DIFFRAC-EVA.V4.2 software and the Crystallography Open Database (COD). The quantitative XRD findings for OPC and FA are given in Table 2.

**Figure 1:**
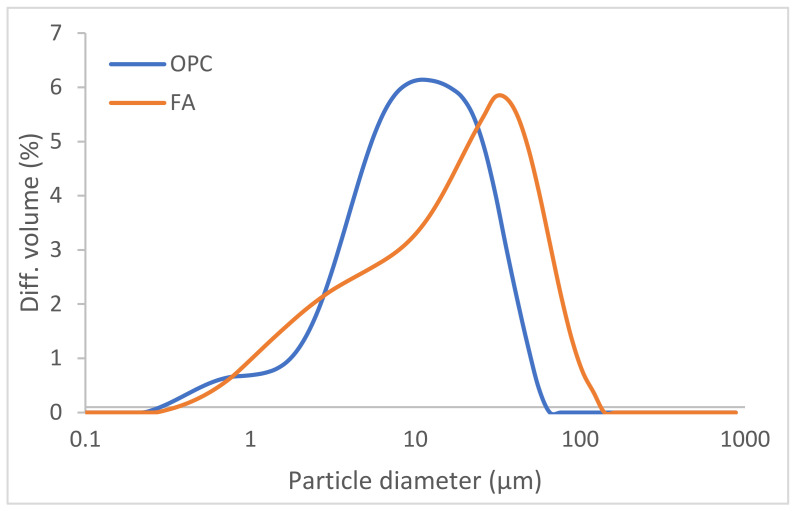
OPC and FA particle size distribution.

OPC and FA particle size distribution, determined by laser diffraction on a Mastersizer analyser (Micromeritics. Norcross, Atlanta, USA) fitted with an He-Ne 632.8 nm laser, is listed in Table 3 and graphed in Figure 1. OPC and FA Blaine fineness were determined as described in European standard EN 196-6:2010 [42].

### 2.2. Anhydrous Hybrid Cement (30% OPC + 70% FA) and Hydrated Paste Preparation

The two hybrid cement components, 30 wt% OPC and 70 wt% FA, were blended in a mechanical shaker-mixer for 90 min to ensure homogeneity. The hybrid cement pastes were prepared varying the parameters as described below:

1. Nature of the alkaline activator: sodium hydroxide (NaOH=N) or sodium hydroxide + hydrated sodium silicate, commonly called waterglass (NaOH + WG = N-WG). The NaOH (Scharlab,, Spain) pellets used were 98% pure; the Na_2_SiO_3_ (Merck, Germany), which contained 25.5% SiO_2_, 7.5% Na_2_O and 67% H_2_O, had a density of 1.365 g cm^−3^.
2. Reaction time: 2 days or 28 days.
3. Curing temperature: (a) curing at 80 °C for 20 h plus chamber curing at 21 ± 2 °C and 99% RH; or (b) humidity chamber curing only from the outset at 21 ± 2 °C and 99% RH.

**Figure 2:**
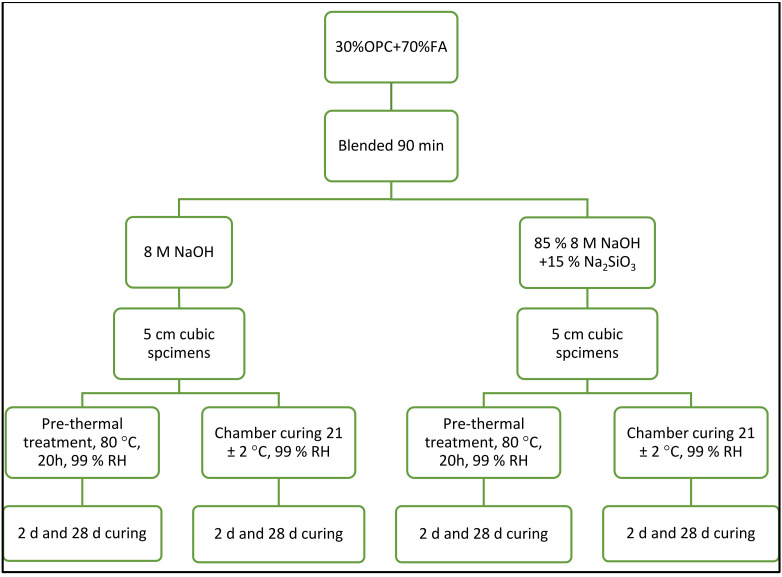
Flowchart for procedure followed and variables used to prepare hybrid cement paste specimens.

The flowchart for the process followed to prepare hybrid cement-activated pastes and the variables used are depicted in Figure 2. The 5 cm specimens were prepared as specified in European standard EN 197-1:2018 [43]. The L/S ratio used was as required to ensure consistency was the same in all samples and as defined in European standard EN 196-3:2017 [44]. The nomenclature assigned the pastes prepared for the study is listed in Table 4.

### 2.3. Tests Conducted

Hybrid cement activation/hydration was detained in the 2 days’ and 28 days’ samples prior to mechanical, physical-chemical, mineralogical, microstructural and radiological characterization by soaking the specimens in isopropanol for 48 h [45]. The samples were subsequently dried under an infrared lamp for 5 min and stored in a vacuum desiccator until reaching a constant weight to eliminate moisture and any remains of isopropanol. The tests described below were conducted on the dry specimens.

#### 2.3.1. Compressive Strength and Mean Pore Size

Compressive strength was found on an Ibertest Autotest 15000 kN test frame as described in European standard EN 196-2:2014 [39]. The value given is the mean for three specimens per paste and curing age. Paste mean pore size was determined on a Micromeritics Autopore IV 9500 Hg intrusion porosimeter designed for pressures of up to 32,000 psi, equivalent to a pore size of 0.0067 µm.

#### 2.3.2. Paste Mineralogical and Microstructural Characterization

The crystalline and semi-crystalline phases present in the hybrid cements and pastes were identified by X-ray diffraction on the diffractometer described in Section 2.1. The diffractograms were recorded with Cu-Kα_1_,α_2_ radiation across the (2θ) 5° to 60° range at a step size of 0.01981° and a step time of 0.5 s.

The reaction of activation products formed were quantified with differential thermal and thermogravimetric (DTA/TG) analysis on a TA Instruments Q600 TGA-DSC-DTA analyser (New Castle, DE, USA). The thermograms were recorded initially from 25 °C to 80 °C and subsequently at a constant 80 °C for 1 h to ensure all the unbound water was released. The samples were then ramped at a rate of 10 °C min^−1^ to 1000 °C in a 100 mL min^−1^ flow of N_2_.

System microstructure was characterised with a Jeol JSM-500 tungsten filament backscattered scanning electronic microscope (BSEM) close-coupled to an Oxford Link-ISIS-EDX energy dispersion X-ray (EDX) spectrometer. The samples were sealed with epoxy resin, polished and their surfaces carbon-coated with a Quorum 150T ES sputter coater to ensure conductivity. The chemical composition of the hybrid cement pastes was determined by EDX-analysis of 40 to 45 points on their respective matrices.

The structure of the alkali-activated materials was studied with high-resolution, solid-state ^29^Si and ^27^Al MAS-NMR spectroscopy. The spectra were collected (at 79.49 MHz and 104.23 MHz, respectively) on a Bruker Avance 400 spectrometer fitted with a Fourier transform unit and employing a 9.4 T magnetic field. The samples were spun at the magic angle (54°44′) at 10 kHz. The pulse widths were 6 µs in ^29^Si and 2 µs in ^27^Al MAS-NMR and the recycle delay times were 10 s and 5 s, respectively, to maximise experimental signal intensity. A total of 800 accumulations were performed for the Si and 200 for the Al signals. The chemical shifts δ (in ppm) given are relative to tetramethylsilane (TMS) for ^29^Si and to Al(H_2_O)_6_^3+^ for ^27^Al. Spectra were fitted to Gaussian and Lorentzian peak shapes using Winfit software by Bruker, based on non-linear least-squares iteration.

#### 2.3.3. Radiological Characterization of the Hardened Intact Hybrid Cement Pastes and Solid Powder Samples

The materials used in this study were characterized with the HPGe gamma spectrometry technique endorsed in Spanish, European and international standard UNE EN ISO/IEC 17025:2017 [46]. Two of the three detectors used were n-type coaxial closed-end instruments and the third a broad energy analyser made of a p-type material (Supplement S1, Table S1). Canberra Industries detectors, high voltage power sources, amplifiers, ADCs and AIMs were used, whilst Genie 2000 software (Canberra Industries, Meriden, Connecticut), also by Canberra Industries, was used for spectrum acquisition and analysis. The following gamma emitters were analysed (Be, 2000):^234^Th (63.30 (2) keV), ^226^Ra (186.211 (13) keV), ^214^Pb (351.932 (2) keV), ^214^Bi (609.312 (7) keV, 1120.287 (10) keV, 1764.494 (14) keV), ^210^Pb (46.539 (1) keV, ^212^Pb (238.632 (2) keV), ^208^Tl (583.187 (2) keV, ^228^Ac (911.196 (6) keV), ^235^U (163.365 (3) keV, 205.16 (4) keV, 143.767 (3) keV) and ^40^K (1460.822 (6) keV). ^235^U interference in the ^226^Ra 186 keV photo peak and ^228^Ac interference in the 1460 keV ^40^K photo peak were suppressed by applying the algorithm developed in [47].

The efficiencies required to determine activity were calculated for two geometries [12]: (i) a 76 mm, 30 mm high cylindrical plastic container for the solid powder and liquid samples; and (ii) 5 cm cubic specimens sealed with epoxy resin as described in an earlier paper [13]. The materials radiologically characterized in this study are listed below:

(a) Anhydrous 30%OPC + 70%FA powder samples with a particle size of under 63 µm dried to a constant weight, stored for 21 days in the aforementioned sealed plastic container to ensure secular equilibrium and subsequently measured in the same container. The activity concentration for each gamma emitter in the natural radioactive series, corrected for their L/S ratio, was denominated ‘calculated activity’.
(b) Hardened (30%OPC + 70%FA) hybrid cement pastes prepared with different alkaline activators and molded in 5 cm cubic specimens, cured under the two temperature regimes specified above for 2 days or 28 days.
(c) Solid hybrid cement (30%OPC + 70%FA) paste powder, ground from the cubic specimens described in b) to a particle size of 63 µm and packed in cylindrical plastic containers for analysis.
(d) The liquid solutions, likewise in cylindrical plastic containers, used to prepare the hardened hybrid cement pastes: (a) water; (b) waterglass and (c) 8 M NaOH.

The activity concentrations for the gamma-emitting radionuclides in the natural radioactive series for uranium, thorium and actinium, along with ^40^K, obtained for the type b and c samples were compared to the ‘calculated activity’ (type a samples) based on their correlation for a coverage factor of k = 2.

^226^Ra activity concentration was also determined radio-chemically on four powder samples (type c). Aliquot samples weighing 0.5 g each were selected and mineralized in an Ethos One microwave oven using a mix of (9 mL) HNO_3_ and (3 mL) HF. The Ra was separated from the resulting solution with a method based on BaSO_4_ precipitation using Ba^2+^ and Pb^2+^ carriers, as described in [48]. The (Pb-Ba)SO_4_ precipitate initially formed was dissolved in an EDTA solution in a medium with a high ammonia content. The BaSO_4_ was then re-precipitated with acetic acid at pH = 4.5, which purified the alpha emitting elements present in the sample. The BaSO_4_ precipitate was filtered and measured in a Canberra Industries ZnS (Ag) solid scintillation detector.

The final activity concentrations were determined from the weighted mean of the individual values (Equation (2)). The uncertainty associated with activity concentration was calculated further to the Bambyneck [49] criterion, i.e., as the higher of the values calculated from Equations (3) and (4),

(2) $$A=\frac{\sum_{i=1}^{N}\left(A_{i}\times u\left(A_{i}\right)\right)}{u\left(A_{i}\right)}$$

(3) $$u{\left(A\right)}_{ext}=\sqrt{\frac{\sum_{i=1}^{N}u{\left(A\right)}_{i}\cdot {\left(A_{i}-A\right)}^{2}}{\frac{N}{N-1}\cdot \sum_{i=1}^{N}u{\left(A\right)}_{i}}}$$

(4) $$u{\left(A\right)}_{int}=\sqrt{{\left(\sum_{i=1}^{N}u{\left(A\right)}_{i}^{-2}\right)}^{-1}}$$

where $A_{i}$ is the i-th activity concentration and $u{\left(A\right)}_{i}$ is the associated uncertainty.

The emanation factor for ^222^Rn (*ε*) in the solid powders was calculated from the equation proposed by Pakou et al. [50]:

(5) $$\varepsilon =\frac{A_{226_{Ra}}-A_{214_{Pb}}}{A_{226_{Ra}}}$$

where $A_{226_{Ra}}$ is the radiochemically determined activity concentration for ^226^Ra and $A_{214_{Pb}}$ is activity concentration for ^214^Pb determined with gamma spectrometry from the 351 keV photopeak.

## 3. Results and Discussion

The most prominent findings stemming from this study are discussed in this section, which analyses the effect of the three variables used on the mechanical strength, minerology and structure of the hybrid cement pastes and on their radiological behavior. The relationship between the microstructure generated in the pastes and their radiological content is also verified. Lastly, the validity of the new gamma spectrometric method to measure these hybrid cement pastes in intact (5 cm cubic), as compared to ground, specimens is assessed.

### 3.1. Hybrid Cement Paste Compressive Strength and Mean Size Pore

Further to the values listed in Table 5 for the hybrid cement pastes, the 2 days and 28 days HN T80 pastes exhibited the greatest compressive strength, followed by the 2 days and 28 days H-WG T80 specimens. Curing at 80 °C for 20 h may consequently be deemed to enhance short-term strength development, accelerating cement hydration and fly ash dissolution [51,52]. It is well known that the thermal curing of alkaline-activated pastes, mortars and concretes induce high mechanical strengths at early ages that are then not affected (increased) with time. This is what happens in the HN-T80-2 and HN T80-28 pastes. The differences in compressive strengths observed, considering the standard deviations, are practically the same, confirming that the strengths obtained at early ages (2 days) does not vary with time (28 days). This explains the similar values obtained in the mean pore size of these pastes. That effect was also observed by other authors [53,54,55,56]. The lowest strength was found for the HN T25-2 pastes, although when these same specimens were cured for 28 days (HN T25-28) they developed nearly the same strength as observed in the highest performing HN T80-28 material. That effect, recorded in all the pastes that were not thermally cured, attested to the continuation over time of the hydration and activation reactions at ambient temperature.

The nature of the activator also affected early-age strength development at ambient temperature. The H-WG T25-2 pastes exhibited greater strength than the HN T25-2 pastes due to the extra soluble silica (sourced from the WG) in the medium and the early formation of reaction product [53,57,58]. As expected, an inverse relationship was observed between compressive strength and mean pore size (see Table 5), a finding consistent with earlier reports [59,60,61].

### 3.2. Hybrid Cement Paste Mineralogical and Microstructural Characterisation

The X-ray patterns for the raw materials (OPC and FA) and the hybrid pastes are reproduced in Supplementary Materials (Appendix 2, Figure S1). The X-ray pattern for the anhydrous OPC contained diffraction lines for the crystalline phases typical of Portland cement: alite (2θ = 29.36°, 32.15°, 32.54°, 41.23°, 51.68°), belite (2θ = 31.00°, 32.12°, 32.54°, 39.25°, 41.20°), tricalcium aluminate (2θ = 33.23°, 47.65°, 59.59°), tetracalcium aluminate ferrite (2θ = 12.22°, 23.11°, 33.94°), gypsum (2θ = 12.14°, 20.96°) and calcite (2θ = 23.10°, 29.41°). Line intensity declined and some signals vanished altogether (gypsum, tricalcium aluminate, ferrite) as cement hydration progressed. The FA diffractogram exhibited signals for quartz (2θ = 20.82°, 26.60°, 45.70°, 50.08°), mullite (2θ = 16.38°, 25.95°, 26.21°, 30.23°, 33.17°, 39.21°, 40.81°) and hematite (θ = 24.13°, 30.92°, 33.17°, 49.43°, 53.90°), as well as an amorphous hump spanning 2θ angles 25° to 30°, characteristic of the non-crystalline fraction in this material. With the reactions, the hump shifted to higher 2θ values (25° to 40°), corroborating FA activation and (N,C)-A-S-H-like gel formation [62,63,64].

Of the variables studied here, curing time had the heaviest impact on paste crystalline phase composition. Zeolite-like phases were identified with the two activators used (N and N-WG) in both the 2 d and the 28 d materials (see Supplementary Material, Figure S1), namely: hydroxysodalite (Na_4_Al_3_Si_3_O_12_OH) at 2θ = 14.04°, 24.48°, and chabazite-Na (NaAlSiO_6_.3H_2_O) at 2θ = 9.30°, 12.78°, 17.42°, 20.45°, 30.38°. Hydroxysodalite content remained practically invariable over time, in contrast to chabazite-Na content, which grew. Similar results were reported by other authors [65,66]. Zeolite phases were not detected in the hybrid systems cured at ambient temperature.

Minority phases were identified in all the pastes, including: calcite (2θ = 23.10°, 29.41°), natron (Na_2_CO_3_·10H_2_O) (2θ = 16.31°, 30.81°) and nahcolite (NaHCO_3_) (2θ = 30.11, 34.30°), all resulting from partial sample weathering [67,68,69,70]. The presence of a semi-crystalline, C-A-S-H-like gel cannot be ruled out, however, for its highest intensity diffraction line (2θ = 29.5°) overlaps with the main calcite diffraction line (2θ = 29.4) [64,71]. Neither portlandite (Ca(OH)_2_) nor ettringite was detected in any of the pastes, a finding likewise consistent with earlier studies [72].

The TG-determined weight loss for all the hybrid pastes is shown in Table 6, while their thermograms are reproduced in Supplementary Materials (Appendix 2, Figure S2).

The temperature ranges shown in Table 6 are based on the physical–chemical changes observed in the hybrid paste thermograms. The samples were kept at 25 °C to 80 °C until their weight was constant to eliminate all bound water [73]. The main reaction products, (N,C)-A-S-H, C-S-H and C-A-S-H gels [74,75], formed during hydration / activation, along with the zeolites (hydroxysodalite and herschelite) detected with XRD [76,77], decomposed in the first range (80 °C to 350 °C). The data also denoted a very close correlation between compressive strength and weight loss detected in this temperature range: the greater the loss and therefore the greater the amount of reaction product forming, the higher the compressive strength (see Table 6). The portlandite forming with cement silicate hydration [78] dehydroxylated in the second temperature range, 350 °C to 500 °C, but were detected only in the ATD/DG thermograms for the pastes activated and cured at ambient temperature (see Table 6 and Supplement 2, Figure S2c,d,g,h). That reaction product was not detected with XRD, very likely because the amount of the mineral formed was too small to be detected by this technique. The carbonates Na_2_CO_3_, NaHCO_3_ [67] and the crystalline and amorphous calcium carbonates [79,80] present in the samples decomposed in the third and last thermal range, 500 °C to 1000 °C. Further to the findings delivered by this technique (Table 6 and Supplementary Material Figure S2), weight loss in the first temperature range, the one associated with reaction product decomposition, grew with reaction time, denoting an ongoing mix of hydration and geopolymerization [81,82,83,84]. Weight loss was greater in Na(OH)-activated pastes (labelled N pastes) than in the ones activated with waterglass (labelled N-WG), attesting to a greater amount of reaction product in the former.

The BSEM micrographs for all the hybrid pastes are shown in Supplementary Materials (Appendix 2, Figure S3). Anhydrous or partially reacted OPC and FA particles were clearly visible (thanks to their distinct morphology, polyhedral in OPC and nearly spherical in FA) on all the images. The cementitious matrix, consisting in much smaller, darker particles, occupied the rest of the space. An analysis of the micrographs in Figure S3 in Supplementary Material: showed that: (i) the N-activated pastes (Supplementary Material, Figure S3a–d) had a denser microstructure than the ones activated with N-WG (Supplementary Material, Figure S3e–h; (ii) reaction time also affected microstructure, raising density and lowering the presence of anhydrous phases; and (iii) thermal curing induced a denser microstructure (irrespective of the activator and reaction time).

**Figure 3:**
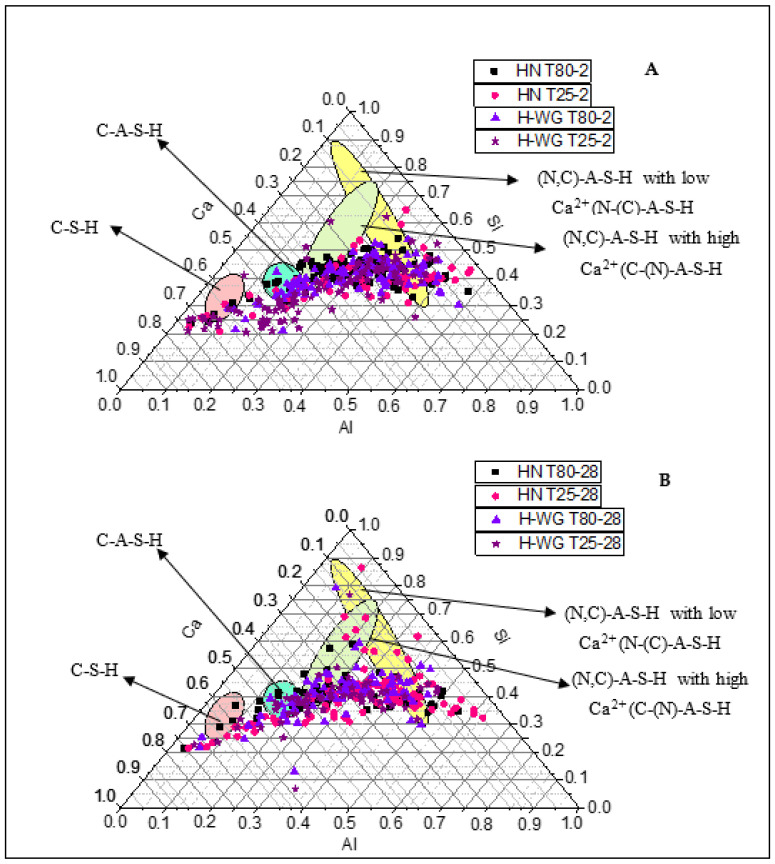
Ca-Si-Al ternary diagrams for (**A**) 2 days and (**B**) 28 days of HN T25, HN T80, H-WG T25 and H-WG T80 pastes.

The information on the composition of the main reaction products in these hybrid pastes obtained with EDX analysis of the areas comprising the cementitious matrix is translated into the Ca-Si-Al ternary diagrams in Figure 3, plotted as atomic percentages normalized to 100%. The color code for the reaction products depicted on the figure is as follows: C-S-H gel (pink); C-A-S-H gel (blue); Ca-high (C,N)-A-S-H [(C-(N)-A-S-H-like)] gel (green); and Ca-low (C,N)-A-S-H [(N-(C)-A-S-H-like)] gel (yellow).

The findings showed that the reaction products generated were practically the same irrespective of the variables used in this study. Nonetheless, this technique shows the composition although not the amount of product forming (found with other methods to differ depending on the variable used). The C-S-H gel (pink) identified, with a Ca/Si ratio of around 2.5, was attributed mainly to Portland cement hydration reactions [85]; the C-A-S-H gel (blue) with a ratio of 1.1 ≤ Ca/Si ≤ 1.3, in line with [17,64,86,87], was the reaction product resulting from the presence of Al in the medium, sourced from the fly ash and the calcium aluminates in the cement. Mixes of (N,C)-A-S-H gels were also observed: in those with high Ca^2+^ content ((N)-C-A-S-H) Ca/Si and Al/Si were both 0.6 whereas in those with low Ca^2+^ (N-(C)-A-S-H), Ca/Si was 0.2 and Al//Si was 1. The two Ca/Si ratios are consistent with the values reported in [88,89,90], whilst the Al/Si ratios are similar to the findings published in [91].

Hybrid cement paste structures were characterized with solid-state NMR, specifically using ^29^Si and ^27^Al nuclei. The deconvoluted data for the 2 days ^29^Si MAS-NMR spectra are given in Table 7 and for the 28 days’ materials in Table 8. The deconvoluted spectra are reproduced in Supplementary Materials (Appendix 2, Figures S4 and S5).

The ^29^Si MAS-NMR spectrum for the anhydrous OPC could be deconvoluted into four signals indicative of tetrahedrally coordinated Si (SiO_4_^4−^ Q^0^ monomers) [92,93,94], attributable to the anhydrous phases alite and belite present in OPC. The signal at around −71 ppm and a low intensity signal at −74 ppm were generated by belite (which can be likened to β-C_2_S) and two wider signals at approximately −70 ppm and −73 ppm by alite (C_3_S). The ^29^Si MAS-NMR spectrum for anhydrous FA contained six signals at approximately: −89 ppm attributable to the Q^3^ units in mullite; −97 ppm to Q^3^(3Al) units [95]; −104 ppm to Q^4^(1Al) units; and at −110 ppm and −118 ppm to the Q^4^(0Al) units in the quartz present in fly ash [96]. The wider sixth signal, appearing at around −106 ppm, denoted the presence of a vitreous phase. The spectrum for the anhydrous blend of OPC and FA exhibited two areas, each bearing the signals described for mix components. Given the high FA content in the blend (70%), the ^29^Si spectra were heavily impacted by the paramagnetic interaction with iron. The signal/noise ratio was consequently low, with wide signals and intense spinning sidebands that masked the actual isotropic chemical shift tensor, resulting in inaccurate quantification.

The ^29^Si MAS-NMR spectra for the hybrid cement pastes cured at different temperatures and reaction times, also affected by the paramagnetic interaction with iron, exhibited three distinct albeit overlapping areas. The one on the left could be fitted to the components in the −70 ppm to −74 ppm range, given the presence of Q^0^ monomers attributable to the anhydrous silicates alite and belite present in OPC [17,88].

The Q^1^ units located between −78 ppm and −80 ppm, attributed to the end site in the tetrahedral silicate chain in C-S-H gel, were found in the middle zone. A signal was likewise observed at −85 ppm, generated by the Q^2^_B_ units in the C-S-H gel [97]. The Q^2^(1Al) units located at around −80 ppm to −83 ppm denoted the precipitation of an Al-bearing C-S-H gel in which Al_T_ replaced Si_T_ in a bridging site, indicating the presence of a C-A-S-H-like gel [17,98,99,100,101]. The band at around −88 ppm was associated with Q^3^(1Al) units. The wide, low-resolution signals in the right-side area of the spectrum were due primarily to unreacted FA. Q^4^(mAl) (m = 4, 3, 2, 1 or 0) units also found in this zone were attributed to Ca^2 +^ -low N-A-S-H and (N,C)-A-S-H gels (N-(C)-A-S-H-like gels) [98,102,103], characterized by a highly polymerized aluminosilicate structure in which Si and Al were tetrahedrally coordinated. The signals at around −93 ppm were associated with Q^4^(3Al) signals generated by N-A-S-H gels with reactive and highly polymerized aluminate phases. The Q^4^(2Al) units located at around −97 ppm and Q^4^(1Al) units at around −103 ppm were the most stable Si-high N-A-S-H sites. The signals at −105 ppm and −116 ppm were attributed to Q^4^(0Al) units associated with the presence of the crystalline quartz phases in fly ash [86,102,104].

In light of the overlap between FA and N-A-S-H gel signals and the high noise level, the components in this right-hand area of the spectrum are difficult to assign to the signals observed with any accuracy. Inasmuch as the size of this area declined over time (from 2 days to 28 days), it might be more suitably attributed to FA, although possible N-AS-H formation cannot be ruled out. The middle zone associated with C-A-S-H and (N,C)-A-S-H gels, in contrast, was observed to grow with reaction time.

Given the nature of the mix (high iron content due to the 70% FA), accurate assessment of activator behavior in the pastes prepared is a challenging endeavor. The anhydrous cement signals on the left widened with reaction time due both to amorphization and the paramagnetic medium created by the iron in the ash, with adverse effects on quantitative accuracy in this area. The zone on the right contained new signals attributed to N-A-S-H gel that overlapped with the wide fly ash signals. The following observations may be drawn from the sum of the middle area components associated with C-S-H and/or C-A-S-H gels and those on the right attributed to N-A-S-H gel.

- In all cases, the spectra showed a substantial decline in the anhydrous fly ash signal, including the area that overlaps with the N-A-S-H gel signals.
- The reactions were faster in the pastes prepared with N-WG, as attested to by the scant differences in the spectra over time.
- In the N pastes, the FA signals declined with reaction time.

The ^27^Al MAS-NMR spectra for the anhydrous cement and fly ash are reproduced in Supplementary Material (Supplement 2, Figure S5). The spectrum for the anhydrous cement exhibited two bands at around 64 ppm and 80 ppm attributed to the tetrahedrally coordinated Al in C_3_A and C_4_AF and the aluminum present in the solid alite solution (C_3_S) [93,94,105,106]. The signals observed at 0.28 ppm and 9.72 ppm were generated by the octahedrally coordinated Al units likewise present in C_4_AF. The ^27^Al MAS-NMR spectrum for the anhydrous fly ash contained two signals, one centered at 59.09 ppm, associated with tetrahedrally coordinated aluminum and the other, a smaller band, at -1.84 ppm, associated with octahedral aluminum. The latter was attributed primarily to the presence of mullite [107].

The ^27^Al MAS-NMR spectra for the hybrid cement pastes, in turn, had two clearly identified signals: a narrow band at around 58 ppm associated with the Al_T_ both from the unreacted fly ash and the reaction products (Al-bearing C-S-H gel (C-A-S-H-like gel)) and N-A-S-H where Ca was replaced by Na ((N,C)-A-S-H-like gel) [108]; and a signal at 0.25 ppm attributed to the Al_O_ in the secondary reaction products and unreacted Portland cement.

As a rule, the signal associated with the Al_T_ in the 2 days and 28 d alkali-activated hybrid cement pastes shifted to lower ppm values than the anhydrous materials (64 ppm and 80 ppm) [98,100,109], an indication of ongoing hydration and geopolymerization. The signal attributed to Al_O_, in contrast, remained essentially unchanged [17].

A comprehensive analysis of all the findings for hybrid paste characterization revealed that the three variables studied (activator composition, reaction time and curing conditions) affected hybrid cement (30%OPC + 70%FA) reactions, for although the same reaction products formed, the microstructures developed varied.

The EDX and NMR results showed that the nature of the reaction products formed, based on C-S-H, C-A-S-H and (N,C)-A-S-H gels, was very similar in all cases, irrespective of the variable studied. Hydroxysodalite and chabazite-Na-like zeolites were identified with XRD in the thermally cured specimens. The amount of product formed nonetheless varied, as revealed by the amount of chemically bound water loss detected with ATD/TG.

Hybrid cement paste microstructures differed significantly with the values chosen for each variable. N-activated, thermally cured pastes exhibited a denser microstructure than the N-WG-activated materials due to the formation of more reaction product, as inferred from the high mechanical strength values, small mean pore sizes and greater weight loss in the 80 °C to 350 °C range recorded for the former. In the pastes not thermally cured, however, the reactions were favored by the Si in the medium released by the waterglass activator, resulting in greater strength in these than in the non-thermally cured N pastes.

The compressive strength, mineralogical and microstructural characterization findings for the hybrid pastes showed that curing temperature and activation and/or reaction time played an important role. Thermal curing raised the silicate hydration rate in the cement, as well as fly ash reactivity and its dissolution rate. That in turn generated more cement hydration product and geopolymerization and consequently a denser microstructure with a concomitant increase in compressive strength. Longer curing times also enhanced reaction progress. The result was more compact microstructure as shown by the BSEM findings, with a decline over time in the amount of unreacted OPC and FA. That was corroborated by the lower intensity of both the diffraction lines for the characteristic anhydrous OPC and FA phases and the Q^0^ and Q^4^(0Al) ^29^Si MAS-NMR signals recorded for those materials.

### 3.3. Hybrid Cement Paste Radioactivity

The activity concentrations found for the 5 cm cubic hardened hybrid cement specimens and their solid ground powders are listed in Table 9. The bottom row of the table gives the activity concentration of the radionuclides in the 30% OPC + 70% FA blend corrected for the L/S ratio (‘calculated activity’). The correlation observed between the ‘calculated activity’ concentration and the activity concentrations measured in the hardened hybrid cement pastes was 91.2% and somewhat lower for the powder samples: 78.8%. Those findings indicate that varying the type of activator, temperature and curing time did not affect the radioactive content in the specimens, for the differences in the values were not statistically significant. The conclusion that may be drawn from the data in Table 9 is that the new method for determining radionuclides in 5 cm cubic hardened paste specimens is valid for hybrid or alkali-activated cements. This methodology can also be applied in mortars [110].

The lower correlation observed for the solid powder samples was attributable to the activity concentrations found for ^214^Pb and ^214^Bi. Although both radionuclides should have been in secular equilibrium with ^226^Ra after 21 d in those samples as they were in the intact specimens, their ratio was approximately 0.5, irrespective of the parameter studied. For that reason, in four of the samples the activity concentration of ^226^Ra was determined via radiochemical separation but also measured with a solid ZnS(Ag) scintillation detector 7 months after the containers were sealed (Table 10). The disequilibrium observed would be explained neither by diffusion across the container walls or lid nor by the variation in efficiency due to loose packing [111,112,113], for the material prepared was equivalent to the ‘calculated activity’ concentration sample, characterized by secular equilibrium between ^226^Ra and both ^214^Pb and ^214^Bi (see Table 10). Furthermore, earlier studies showed fly ash to have a low ^222^Rn emanation fraction (ε%) [114], whereas the values found in this study were high, with a mean of 50%. However, those authors’ findings were for uniform distribution of Ra across the surface of the solid particles [115]. Therefore, C-A-S-H and (C,N)-A-S-H gel formation from the fly ash in hybrid cement activation might be deemed the cause of this structural change in the Ra in the particles studied here (see discussion in the final paragraph of this subsection). That effect was observed only in the ground samples, however, and not in the intact 5 cm cubic specimens, denoting a small mean pore diameter in the end material that would prevent ^222^Rn emanation. Further study would be required to fully explain those observations.

The ACI determined from the mean activity concentrations for ^226^Ra (94.1 ± 4.8 Bq kg^−1^), ^212^Pb (^232^Th) (39.6 ± 1.5 Bq kg^−1^) and ^40^K (39.6 ± 1.5 Bq kg^−1^) in the end yielded an ACI of 0.5733 ± 0.0096. With such an ACI the hybrid cement pastes could be deemed to be radiologically safe for use as construction materials.

As earlier studies showed that fly ash had higher activity concentrations than Portland cement [116,117], a substantial share of the ^226^Ra activity concentration may be said to be attributable to the former. Further to the present findings, based on both intact 5 cm cubic specimens and their ground powder, the latter emanated ^222^Rn (a daughter to ^226^Ra), for ^214^Pb and ^214^Bi were not in secular equilibrium with ^226^Ra in the powder. That might be explained by the fact that the alkaline activation reactions took place very rapidly, prompting structural deformation in the fly ash with the concomitant non-uniform distribution of ^226^Ra on its surface. That development was not observed in earlier studies by Moreno-Reyes et al. [13] in OPC + FA blends mixed with water. The reason for these differences may lie in two factors: i) the maximum FA content in the earlier cements was 30 wt%, whereas fly ash accounted for 70% of the hybrid cements studied here; and ii) the FA reactions in 70% OPC + 30% FA cements were pozzolanic, i.e., much slower than in the alkaline activation of fly ash (particularly in the thermally cured materials). The inference would be that the structural deformation in the FA in the pastes studied in [13] would have been less intense and no ^222^Rn emanation would have been observed.

## 4. Conclusions

This study addressed the effect of hybrid cement (30% OPC + 70% FA) activation variables such as type of activator, reaction time and thermal curing on the nature of the reaction products forming and their microstructure, as well as the effect of those two factors on paste radioactivity. This is the first study on the radiological behavior of hybrid cement pastes carried out to date.

The primary conclusions drawn around the reaction products and microstructures developing are listed below.

1. The three variables studied affected hybrid cement reactions. Although the reaction products formed were the same, their microstructures were not. The products identified were C-S-H, C-A-S-H and (N,C)-A-S-H. Hydroxysodalite and chabazite-Na-like zeolites were also identified in the thermally cured specimens. The amount of product formed also depended on activation variables.
2. The microstructures developed in the hybrid cement pastes differed significantly with the values chosen for each variable. NaOH-activated, thermally cured pastes exhibited a denser microstructure than the NaOH-WG-activated materials due to the formation of more reaction product, as attested to by the high mechanical strength values and smaller mean pore sizes in the former. In the pastes not thermally cured, however, the reactions were favored by the Si in the medium released by the waterglass activator, resulting in greater strength in the N-WG tan in the non-cured N pastes. Denser microstructures were observed at longer reaction times.
3. Thermal curing raised both the silicate hydration rate in the cement and fly ash reactivity and its dissolution rate. That in turn generated more cement hydration product and geopolymerization.

The new gamma spectrometric measuring method, based on solid 5 cm cubic specimens, was validated for the first time for hybrid or alkali-activated cement pastes. The variables involved in hybrid cement activation, including type of alkaline activator and curing time and temperature, were experimentally shown to have no effect on specimen radioactivity, for the values recorded were equal, statistically speaking.

The powder samples, but not the intact specimens, were observed to emanate ^222^Rn (a daughter to ^226^Ra), for the ^214^Pb and ^214^Bi activity concentrations were not in secular equilibrium with the ^226^Ra value. That might be explained by the fact that the alkaline activation reactions took place very rapidly, prompting structural deformation in the fly ash with the concomitant non-uniform distribution of ^226^Ra on its surface. Further research would be required to explain that finding.

## Figures and Tables

**Table 1:** OPC and FA chemical composition (wt%).

Materials	CaO	SiO_2_	Al_2_O_3_	Fe_2_O_3_	K_2_O	MgO	Na_2_O	P_2_O_5_	SO_3_	TiO_2_	Otros	LoI	I.R	SiO_2_S__ ^(1)^	Al_2_O_3_S__ ^(1)^	SiO_2_R__ ^(2)^
OPC	64.47	20.29	5.67	2.35	0.97	0.84	0.11	0.14	2.91	0.24	0.17	2.97	1.07	-	-	-
FA	4.78	42.44	26.95	18.40	1.53	0.80	0.50	0.20	1.44	1.07	0.03	1.63	7.78	29.14	13.02	39.83

LoI: loss on Ignition; I.R.: Insoluble Residue, ^(1)^ Soluble silica (SiO_2_S__) and alumina (Al_2_O_3_S__), ^(2)^ Reactive silica (SiO_2_R__).

**Table 2:** OPC and FA quantitative mineralogical composition (wt%).

	OPC							
Component	Alite	Belite	Tricalcium aluminate	Ferrite	Gypsum	Basanite	Calcite	
(wt%)	64.2	13.2	9.0	5.8	1.8	1.6	4.4	
	**FA**							
Component	Amorphous phase	Quartz	Mullite	Hematite	Magnesium ferrite	Magnetite	Maghemite	Calcite
(wt%)	69.4	6.4	16.5	1.9	3.2	1.3	0.7	0.5

**Table 3:** OPC and FA particle size distribution and Blaine fineness.

Material	Dv10 (µm)	Dv50 (µm)	Dv90 (µm)	Blaine (m^2^ kg^−1^)
OPC	2.3	9.3	27.0	404.7
FA	1.9	16.1	51.5	451.9

**Table 4:** Nomenclature for hybrid cement pastes and variables considered in their preparation.

Sample	Activator	* L/S	Curing Conditions	Reaction Time (days)
HN T80-2	NaOH 8M	0.41	80 ± 1 °C 20 h, 21 ± 2 °C + 99% RH	2
HN T80-28	NaOH 8M	0.41	80 ± 1 °C 20 h, 21 ± 2 °C + 99% RH	28
HN T25-2	NaOH 8M	0.41	21 ± 2 °C + 99% RH	2
HN T25-28	NaOH 8M	0.41	21 ± 2 °C + 99% RH	28
H-WG T80-2	85%NaOH + 15%Na_2_SiO_3_	0.36	80 ± 1 °C 20 h, 21 ± 2 °C + 99% RH	2
H-WG T80-28	85%NaOH + 15%Na_2_SiO_3_	0.36	80 ± 1 °C 20 h, 21 ± 2 °C + 99% RH	28
H-WG T25-2	85%NaOH + 15%Na_2_SiO_3_	0.36	21 ± 2 °C + 99% RH	2
H-WG T25-28	85%NaOH + 15%Na_2_SiO_3_	0.36	21 ± 2 °C + 99% RH	28

* L/S = liquid/solid ratio.

**Table 5:** Hybrid cement paste compressive strength (MPa) and mean pore size (µm).

Samples	Compressive Strength (MPa)	Mean Pore Size (µm)
HN T80-2	50.0 ± 0.7	0.012
HN T80-28	48.8 ± 0.8	0.013
HN T25-2	3.2 ± 0.1	0.081
HN T25-28	45.6 ± 0.8	0.013
H-WG T80-2	36.3 ± 0.4	0.017
H-WG T80-28	44.4 ± 1.6	0.015
H-WG T25-2	15.7 ± 0.4	0.050
H-WG T25-28	32.0 ± 0.4	0.020

**Table 6:** TG-mediated weight loss (wt%) in hybrid cements.

Samples	80 °C–350 °C	350 °C–500 °C	500 °C–1000 °C
HN T80-2	5.1	-	3.8
HN T80-28	5.1	-	4.1
HN T25-2	3.1	0.8	3.8
HN T25-28	4.9	0.9	3.3
H-WG T80-2	3.9	-	3.5
H-WG T80-28	4.1	-	3.6
H-WG T25-2	3.3	0.7	3.1
H-WG T25-28	4.4	1.0	2.7

**Table 7:** Signal positions and intensities on deconvoluted ^29^Si MAS-NMR spectra for 2 days hybrid cement pastes.

Sample	Q^0^				Q^1^	Q^2^(1Al)	Q^2^	Q^3^(1Al)	Q^4^(4Al)	Q^4^(3Al)	Q^4^(2Al)	Q^4^(1Al)	Q^4^(0Al)	
HN T80	−70.75ppm I = 10.42%	−	−	−74.38ppm I = 12.25%	−78.87 ppm I = 1.86%	−81.27 ppm I = 4.44%	−83.97 ppm I = 6.37%	−87.95 ppm I = 12.03%	−90.69 ppm I = 2.10%	−93.20 ppm I = 11.10%	−97.86 ppm I = 7.68%	−102.06 ppm I = 6.77%	−106.74 ppm I = 12.28%	−116,83 ppm I = 12.71%
HN T25	−70.66 ppm I = 5.68%	−71.60 ppm I = 2.55%	−73.49 ppm I = 15.05%	−74.90 ppm I = 0.10%	−79.02 ppm I = 5.53%	−81.37 ppm I = 5.69%	−84.38 ppm I = 11.01%	−87.61 ppm I = 12.19%	−90.82 ppm I = 7.12%	−94,60 ppm I = 5.86%	−98,15 ppm I = 3.78%	−101.63 ppm I = 3.64%	−105,39 ppm I = 6.10%	−113,01 ppm I = 15.69%
H−WG T80	−	−71.70 ppm I = 20.65%	−	−74.20 ppm I = 11.64%	−78.70 ppm I = 1.54%	−81.87 ppm I = 7.02%	−85.41 ppm I = 13.46%	−88.40 ppm I = 6.16%	−90.20 ppm I = 4.62%	−93.27 ppm I = 8.32%	−98.09 ppm I = 6.77%	−102.10 ppm I = 3.76%	−105.77 ppm I = 4.72%	−113.01 ppm I = 11.33%
H−WG T25	−	−71.70 ppm I = 15.95%	−	−74.20 ppm I = 10.44%	−77.76 ppm I = 3.31%	−81.44 ppm I = 7.56%	−84.36 ppm I = 11.50%	−87.15 ppm I = 13.89%	−91.16 ppm I = 7.91%	−94.90 ppm I = 5.36%	−98.52 ppm I = 3.97%	−102.84 ppm I = 3.75%	−106.84 ppm I = 6.85%	−113.01 ppm I = 9.51%

**Table 8:** Signal positions and intensities on deconvoluted ^29^Si MAS−NMR spectra for 28 days hybrid cement pastes.

Sample	Q^0^		Q^1^	Q^2^(1Al)	Q^2^	Q^3^(1Al)	Q^4^(4Al)	Q^4^(3Al)	Q^4^(2Al)	Q^4^(1Al)	Q^4^(0Al)
HN T80	−71.05 ppm I = 22.94%	−74.32 ppm I = 8.19%	−78.54 ppm I = 2.27%	−80.92 ppm I = 9.30%	−84.61 ppm I = 18.04%	−87.98 ppm I = 13.36%	−92.34 ppm I = 9.73%	−95.15 ppm I = 3.74%	−98.66 ppm I = 4.44%	−102.50 ppm I = 2.87%	−109.00 ppm I = 5.13%
HN T25	−71.68 ppm I = 19.44%	−74.80 ppm I = 9.13%	−79,09 ppm I = 8,80%	−82.31 ppm I = 15.44%	−85.42 ppm I = 15.49%	−88.62 ppm I = 10.53%	−91,77 ppm I = 4.40%	−94.81 ppm I = 2.86%	−97.20 ppm I = 2.88%	−101.50 ppm I = 2.46%	−107.31 ppm I = 8.59%
H−WG T80	−71.21 ppm I = 17.21%	−74.02 ppm I = 11.86%	−79.10 ppm I = 4.43%	−82.26 ppm I = 12.86%	−85.20 ppm I = 14.78%	−87.66 ppm I = 7.49%	−89.71 ppm I = 8.06%	−93.31 ppm I = 9.23%	−97.64 ppm I = 6.91%	−102.50 ppm I = 3.60%	−109.00 ppm I = 3.58%
H−WG T25	−71.36 ppm I = 18.79%	−74.02 ppm I = 8.65%	−78.32 ppm I = 5.99%	−81.35 ppm I = 10.60%	−84.45 ppm I = 15.14%	−87.20 ppm I = 8.62%	−90.75 ppm I = 6.42%	−94.83 ppm I = 4.00%	−98.59 ppm I = 2.57%	−101.49 ppm I = 3.60%	−109.00 ppm I = 15.62%

**Table 9:** Activity concentrations for gamma emitters in the uranium, thorium and actinium radioactive series in the samples.

Samples	Curing Time (days)	Type	Uranium Series					Thorium Series			Actinium Series	^40^K
			^234^Th	^226^Ra	^214^Pb	^214^Bi	^210^Pb	^228^Ac	^212^Pb	^208^Tl	^235^U	
HN T80	2	Cubic specimens	93.1 ± 5.3	93 ± 10	83.0 ± 4.7	78.3 ± 5.2	64.2 ± 7.5	35.7 ± 2.7	37.8 ± 2.3	13.7 ± 1.0	4.89 ± 0.66	186.9 ± 4.3
		Ground samples	100.0 ± 6.5	96 ± 16	40.7 ± 3.2	37.6 ± 2.7	61.8 ± 4.6	37.1 ± 1.8	38.1 ± 1.8	14.0 ± 1.0	3.3 ± 1.4	208 ± 20
	28	Cubic specimens	97.8 ± 5.3	98.8 ± 6.3	91.7 ± 5.3	85.9 ± 4.8	63.4 ± 4.9	38.54 ± 0.79	40.9 ± 1.7	14.93 ± 0.50	5.33 ± 0.42	178.1 ± 4.3
		Ground samples	103.5 ± 7.4	93.0 ± 5.9	42.5 ± 4.4	38.3 ± 4.2	63.7 ± 4.8	38.6 ± 1.9	39.0 ± 2.1	14.99 ± 0.95	4.58 ± 0.45	192 ± 10
HN T25	2	Cubic specimens	103.2 ± 5.5	*99.1 ± 6.2*	101.6 ± 3.9	96.2 ± 2.2	63.0 ± 4.8	39.72 ± 0.80	42.0 ± 1.7	16.09 ± 0.51	3.49 ± 0.46	212 ± 14
		Ground samples	99.4 ± 8.5	88.5 ± 5.8	50.4 ± 6.9	45.7 ± 5.8	61.7 ± 5.4	37.2 ± 2.1	38.1 ± 1.8	14.39 ± 0.64	2.79 ± 0.84	193 ± 12
	28	Cubic specimens	97.6 ± 5.5	96 ± 14	96.3 ± 7.5	90.6 ± 8.4	62.3 ± 4.9	37.3 ± 2.7	39.5 ± 2.2	14.96 ± 0.78	6.34 ± 0.63	156.7 ± 9.5
		Ground samples	104.1 ± 5.7	91.7 ± 6.2	50.5 ± 1.9	47.3 ± 2.5	64.1 ± 4.8	38.18 ± 0.82	39.8 ± 1.6	15.03 ± 0.50	5.37 ± 0.82	188 ± 15
H-WG T25	2	Cubic specimens	95.8 ± 6.3	85.3 ± 7.7	94.3 ± 6.7	88.7 ± 6.5	64.2 ± 4.9	36.9 ± 2.1	38.3 ± 2.2	14.90 ± 0.88	5.01 ± 0.27	187.9 ± 4.9
		Ground samples	99.0 ± 7.4	83.8 ± 6.8	55.0 ± 4.9	51.0 ± 4.9	59.0 ± 5.0	36.2 ± 2.0	38.6 ± 2.3	14.78 ± 0.75	4.76 ± 0.46	201.7 ± 8.8
	28	Cubic specimens	105.1 ± 6.0	97 ± 11	104.9 ± 4.0	99.6 ± 1.6	64.7 ± 5.0	39.13 ± 0.85	41.4 ± 1.7	16.20 ± 0.55	3.93 ± 0.68	204 ± 15
		Ground samples	96.1 ± 7.0	82.2 ± 5.9	56.9 ± 3.7	51.5 ± 3.5	62.4 ± 4.7	36.1 ± 1.9	38.0 ± 2.2	14.49 ± 0.76	5.59 ± 0.28	180 ± 12
HN T25	2	Cubic specimens	98.5 ± 5.5	*99.9 ± 6.2*	96.5 ± 3.7	91.4 ± 1.8	61.6 ± 4.7	38.08 ± 0.76	40.6 ± 1.7	14.59 ± 0.48	5.55 ± 0.35	185.9 ± 4.4
		Ground samples	98.3 ± 7.2	89.9 ± 8.6	64.9 ± 5.4	60.9 ± 5.7	64.1 ± 4.8	36.8 ± 2.3	39.1 ± 2.2	15.11 ± 0.78	5.3 ± 1.9	191 ± 16
	28	Cubic specimens	96.8 ± 5.0	85.7 ± 8.9	91.4 ± 5.3	85.6 ± 5.5	63.4 ± 4.9	36.0 ± 1.9	37.7 ± 1.9	13.86 ± 0.77	5.06 ± 0.43	182.7 ± 6.7
		Ground samples	98.1 ± 5.8	91.6 ± 6.9	57.0 ± 4.2	53.1 ± 4.6	58.4 ± 4.9	37.4 ± 2.1	39.0 ± 2.4	15.6 ± 1.1	4.20 ± 0.43	188 ± 24
(*) MAIC	−	Powder	104.4 ± 9.3	84.9 ± 7.8	100.6 ± 6.1	93.3 ± 6.2	60.8 ± 6.0	37.2 ± 1.9	38.9 ± 2.2	15.41 ± 0.69	4.50 ± 0.66	196 ± 19

The uncertainties are quoted for a coverage factor k = 2. (*) MAIC (Mean of Anhydrous Materials).

**Table 10:** ^226^Ra, ^214^Pb and ^214^Bi activity concentrations initially determined with gamma spectrometry and 7 months later with radiochemical separation and solid-state ZnS(Ag) scintillation detector measurements.

Samples	^226^Ra (Rq) (Bq kg^−1^)	Initial Gamma Activity Concentration (Bq kg^−1^)			Final Gamma (7 Month) Activity Concentration (Bq kg^−1^)			ε (%)
		^226^Ra	^214^Pb	^214^Bi	^226^Ra	^214^Pb	^214^Bi	
HN T80-28	93.0 ± 7.1	90 ± 11	39.2 ± 8.0	36.6 ± 7.4	104 ± 10	48.0 ± 3.0	44.3 ± 4.0	52.0 ± 7.1
HN T25-2	94 ± 7	86.3 ± 5.5	56.2 ± 5.3	51.0 ± 3.0	82.3 ± 10.0	45.2 ± 10.0	43.6 ± 10.0	57.1 ± 12.3

Uncertainties are quoted for a coverage factor k = 2.

## Data Availability

The data presented in this study and supplementary information available on request from the corresponding author.

## Supplementary Materials

Figure S1: XRD patterns of anhydrous, 2 days and 28 days hybrid cement: (a) HN T 80, (b) HN T25, (c) H-WG T80 y (d) H-WG T25 (A: alite; B: belite; F: tricalcium aluminate; FF: ferrite; Ba: basanite; G: gypsum; Q: quartz; M: mullite; H: hematite; N: natron; Nh: nahcolite; C: CaCO_3_; Ch: chabazite-Na; S: hydroxysodalite, Figure S2: TG (green) and ATD (blue) curves for 2 days and 28 days pastes: (a) HN T80-2; (b) HN T80-28; (c) HN T25-2; (d) HN T25-28; (e) H-WG T80-2; (f) H-WG T80-28; (g) H-WG T25-2; (h) H-WG T25-28, Figure S3: BSEM micrographs for 2 days and 28 days hybrid cement pastes: (a) and (b) HN T80; (c) and (d) HN T25; (e) and (f) H-WG T80; (g) and (h) H-WG T25, Figure S4: ^29^Si MAS-NMR spectra for: (a) anhydrous OPC; (b) anhydrous FA; (c) anhydrous 30%OPC + 70%FA; (d) HN T80-2; (e) HN T80-28; (f) HN T25-2; (g) HN T25-28; (h) H-WG T80-2; (i) H-WG T80-28; (j) H-WG T25-2; (k) H-WG T25-28, Figure S5: ^27^Al MAS-NMR spectra for: (a) anhydrous OPC; (b) anhydrous FA; (c) HN T80-2; (d) HN T80-28; (e) HN T25-2; (f) HN T25-28; (g) H-WG T80-2; (h) H-WG T80-28; (i) H-WG T25-2; (j) H-WG T25-28, Table S1: Specifications for the high purity germanium detectors used in this study.

## Sample Availability

Samples of the compounds are available from the authors.
