# Supplementary material for: Hybrid Cements: Mechanical Properties, Microstructure and Radiological Behavior
Source: Molecules. 2022 Jan 13;27(2):498. doi: 10.3390/molecules27020498 (PMC8781129; doi:10.3390/molecules27020498)
Supplement: Supplementary file 1 [file molecules-27-00498-s001.zip › molecules-1529778-supplementary.pdf]

molecules

Article
Supplementary materials

Hybrid cements. Mechanical Strengths, Microstructure and Radiological Behavior

Ana María Moreno de los Reyes [1], José Antonio Suárez- Navarro [2], María del Mar Alonso [1], Catalina Gascó[2], Isabel Sobrados[3] and Francisca Puertas [1,*]

1 Department of Materials, Eduardo Torroja Institute for Construction Sciences (IETcc-CSIC), 28033 Madrid, Spain; ana.moreno@ietcc.csic.es (A.M.M.d.l.R.); mmalonso@ietcc.csic.es (M.d.M.A.); puertasf@ietcc.csic.es (F.P.)
2 Department of Environment, Environmental Radioactivity and Radiological Surveillance (CIEMAT), Avenida Complutense 40, 28040 Madrid, Spain; ja.suarez@ciemat.es (J.A.S.-N.); ctlngasleon@outlook.es (C.G.)
3 Department of Energy, Environment and Health, Institute of Material Sciences of Madrid (ICMM.CSIC), 28049 Madrid, Spain; isobrado@icmm.csic.es
* Correspondence: puertasf@ietcc.csic.es

Specifications for the HPGe detectors used

Table S1. Specifications for the high purity germanium detectors used in this study

Parameter	Detector 07	Detector 50	Detector 90
**Model**	GXI0022	GR3321	BE50360
**Type**	Extended range coaxial	Reverse electrode coaxial	Broad energy
**Resolution at 1.33 MeV (keV)**	2.04	2.04	1.84
**Relative efficiency at 1.33 MeV (%)**	115.7	35.5	48.0
**Crystal diameter (mm)**	84	58	80
**Crystal length (mm)**	72	60	30
**Outer shielding (cm)1**	15	15	15
**Inner shielding-1st layer (Cu) (mm)**	3.0	-	2.5
**Inner shielding-2nd layer (Zn) (mm)**	-	-	1.5
**Inner space shielding (dm^3)**	25	25	25
**Shielding composition**	Fe	Fe	Pb

Hybrid cement paste (30% OPC + 70% FA) mineralogical and microstructural characterisation: XRD patterns, ATD/TG thermograms, ^{29}Si and ^{27}Al MAS-NMR spectra and BSEM micrographs.

- XRD Patterns

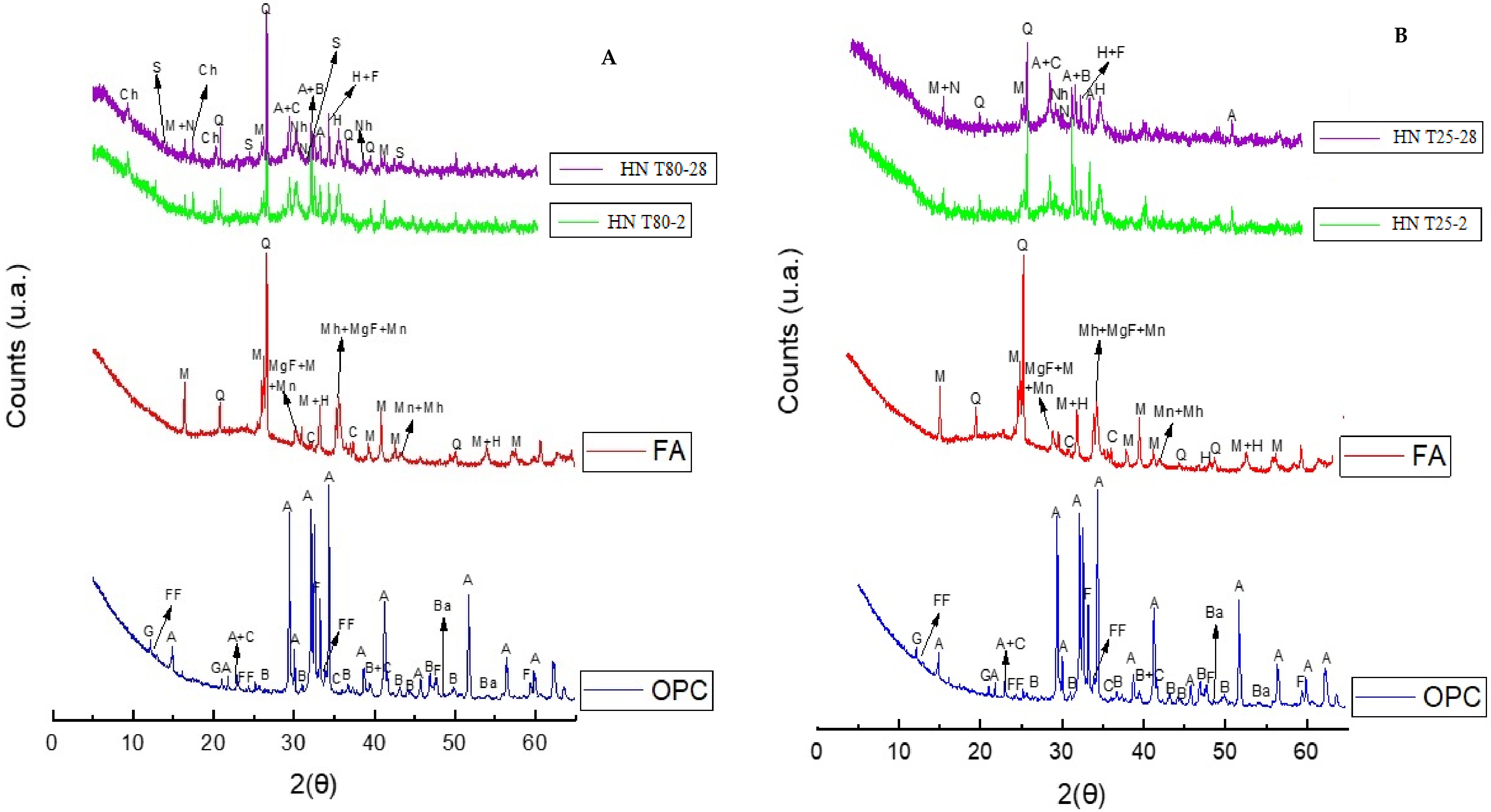

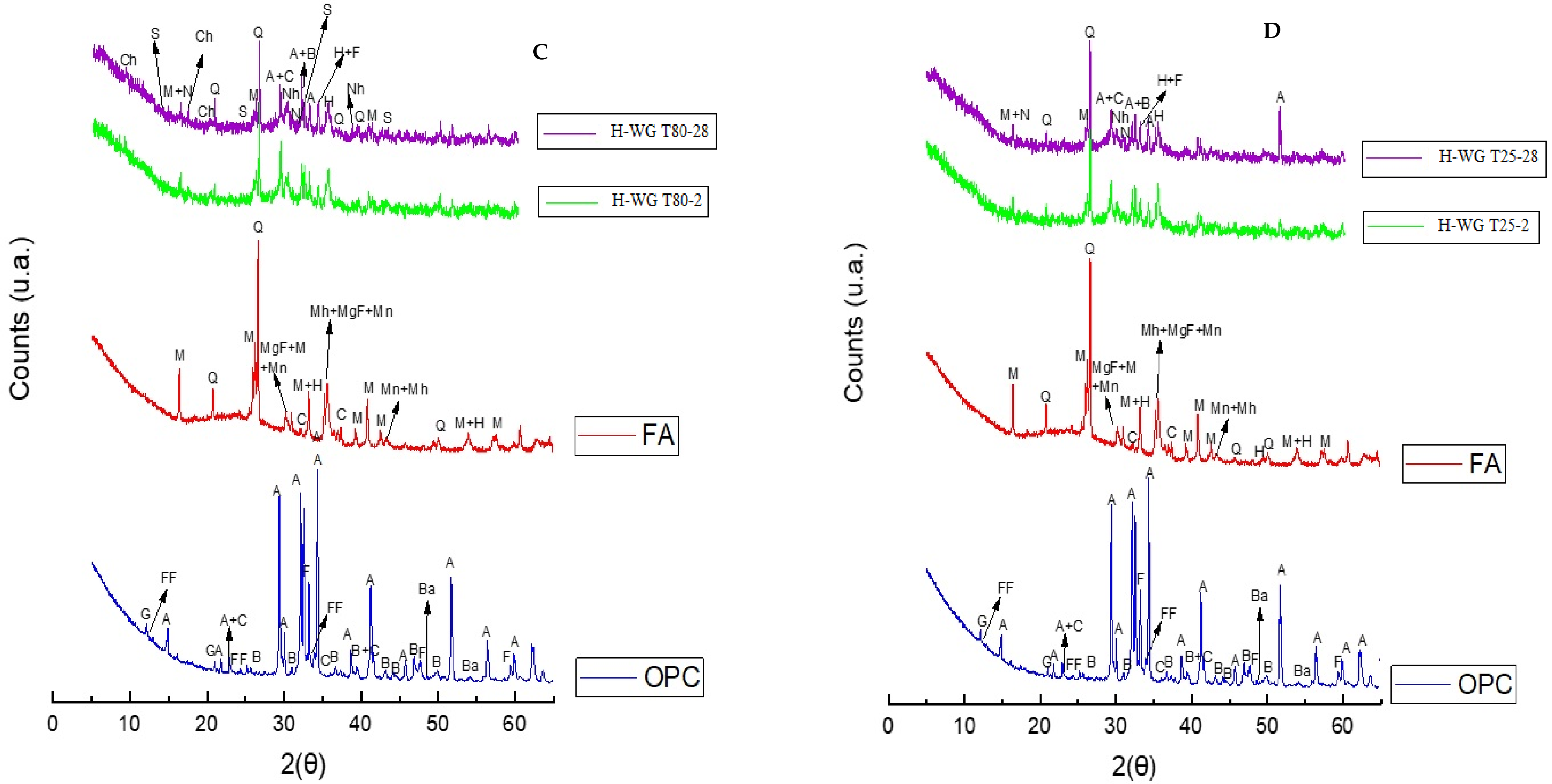

Figure S1. XRD patterns of anhydrous, 2 days and 28 days hybrid cement: (**A**) HN T80; (**B**) HN T25; (**C**) H-WG T80; (**D**) H-WG T25 (A: alite; B: belite; F: tricalcium aluminate; FF: ferrite; Ba: basanite; G: gypsum; Q: quartz; M: mullite; H: hematite; N: natron; Nh: nahcolite; C: $CaCO_3$; Ch: chabazite-Na; S: hydroxysodalite).

- ATD/TG Thermograms

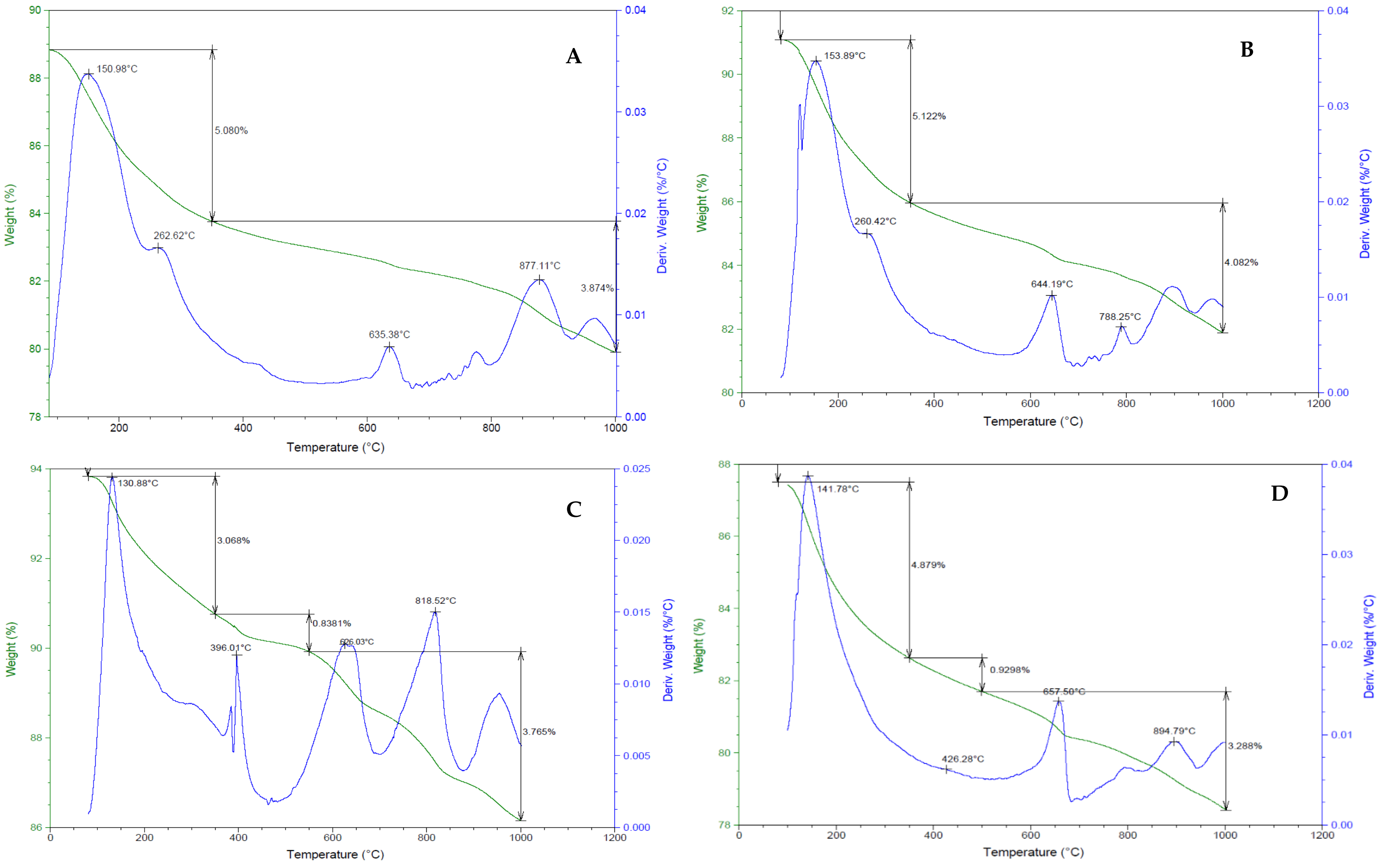

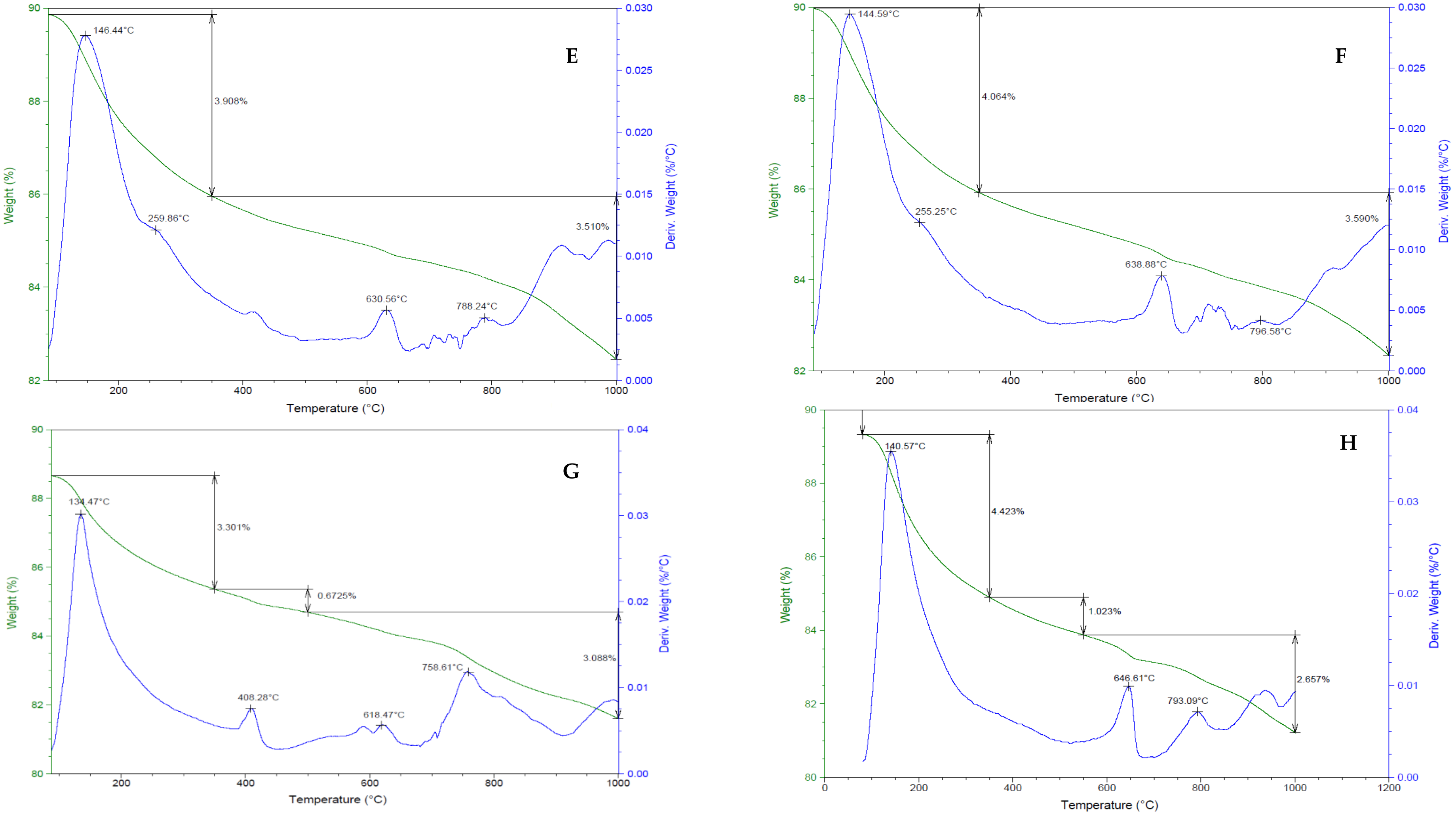

Figure S2. TG (green) and ATD (blue) curves for 2 days and 28 days pastes: (**A**) HN T80-2; (**B**) HN T80-28; (**C**) HN T25-2; (**D**) HN T25-28; (**E**) H-WG T80-2; (**F**) H-WG T80-28; (**G**) H-WG T25-2; (**H**) H-WG T25-28

- BSEM micrographs.

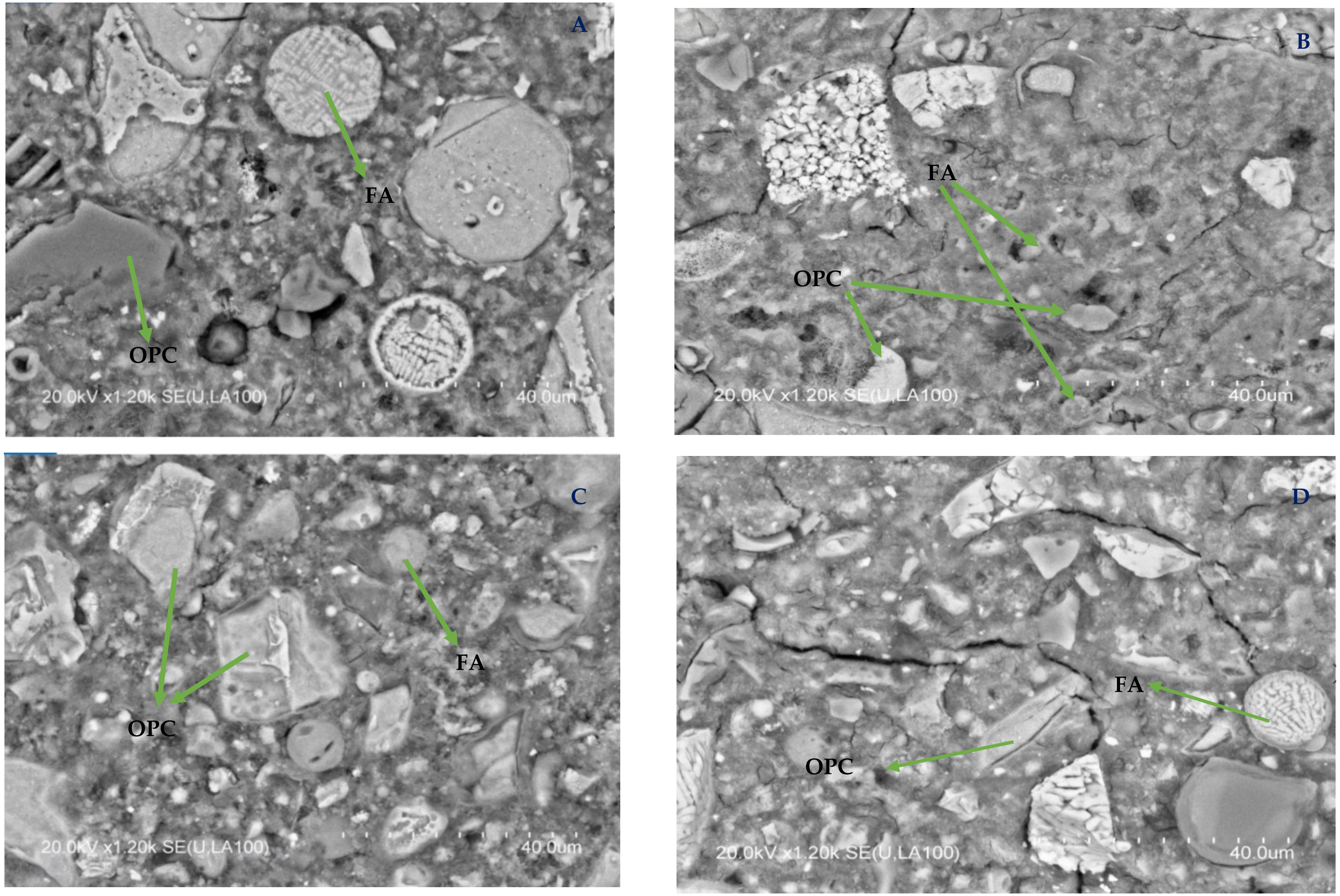

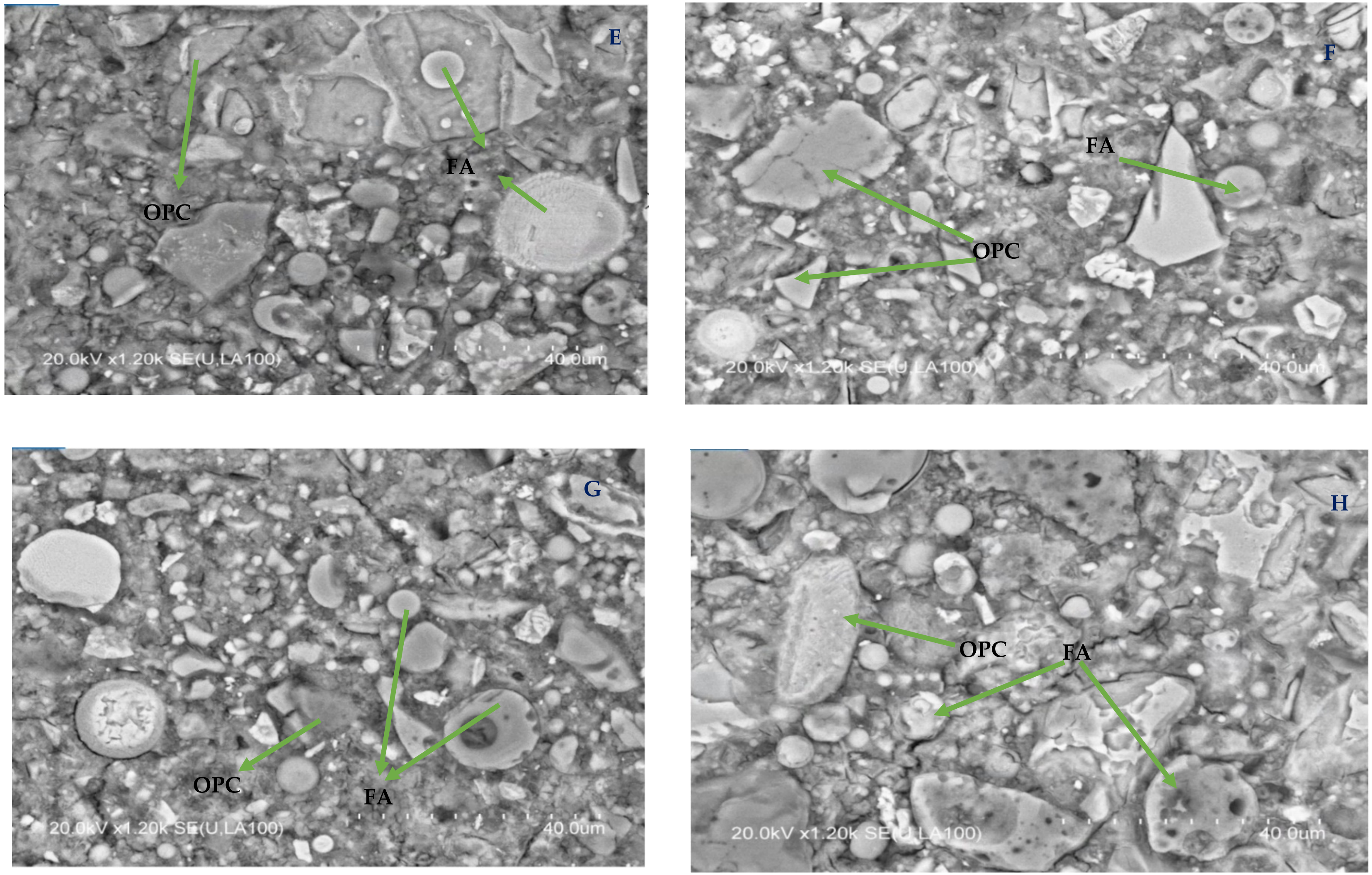

Figure S3. BSEM micrographs for 2 days and 28 days hybrid cement pastes: (**A,B**) HN T80; (**C,D**) HN T25; (**E,F**) H-WG T80; (**G,H**) H-WG T25.

- ^{29}Si MAS-NMR spectra.

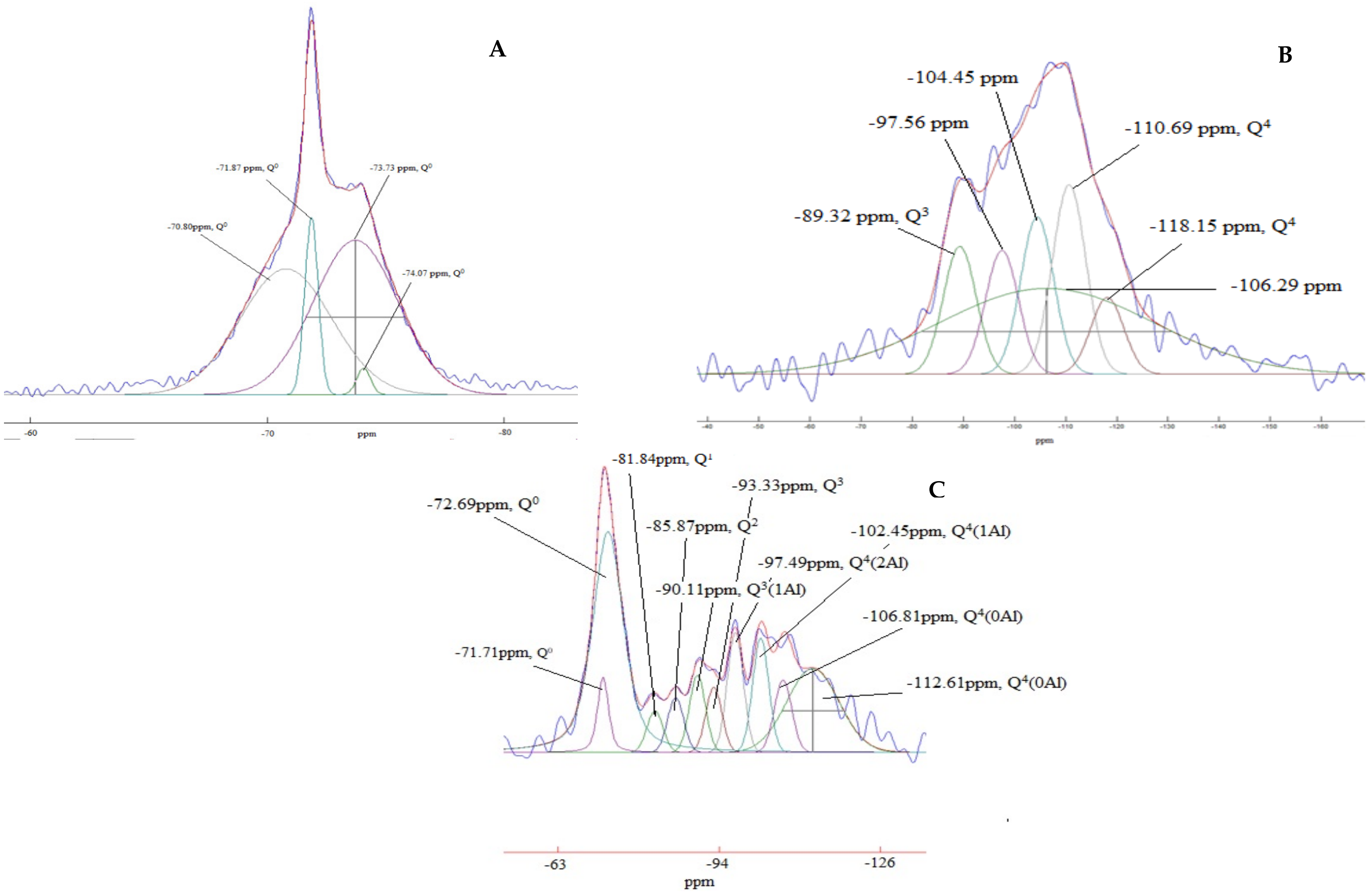

molecules

MDPI

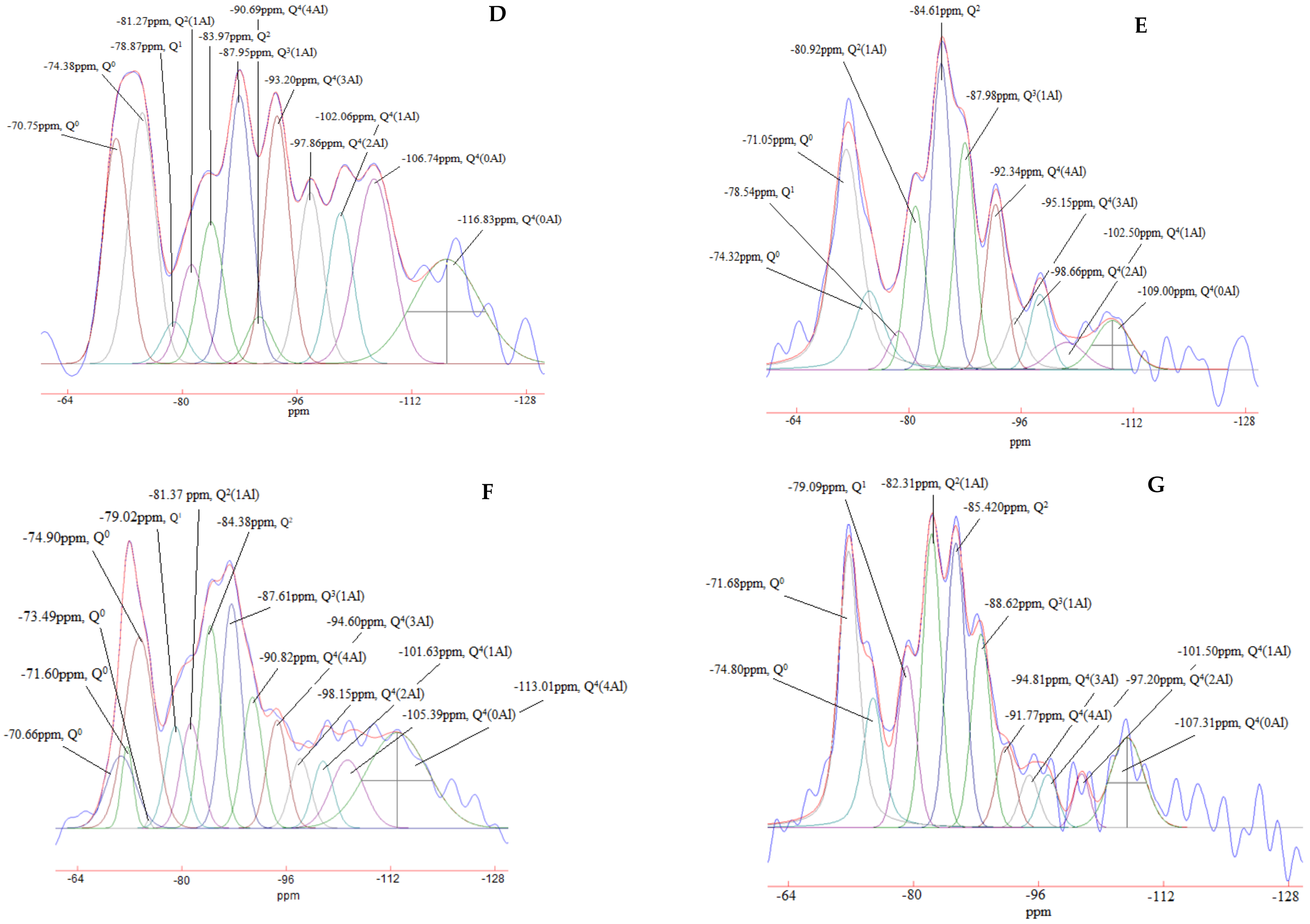

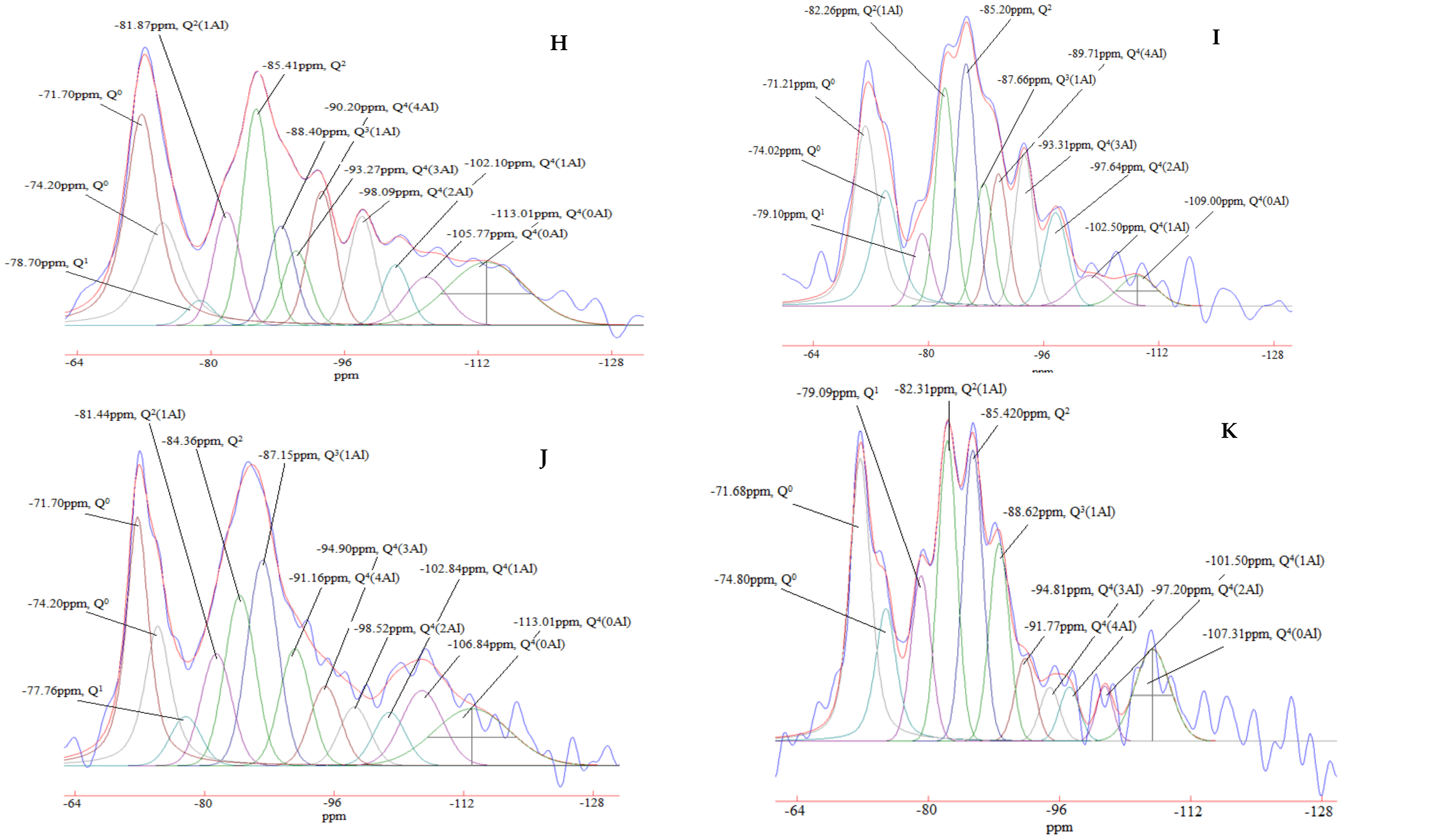

Figure S4. ^{29}Si MAS-NMR spectra for: (**A**) anhydrous OPC; (**B**) anhydrous FA; (**C**) anhydrous 30%OPC+70%FA; (**D**): HN T80-2; (**E**) HN T80-28; (**F**) HN T25-2; (**G**) HN T25-28; (**H**) H-WGN T80-2; (**I**) H-WG T80-28; (**J**) H-WG T25-2; (**K**) H-WG T25-2.

- ^{27}Al MAS-NMR spectra

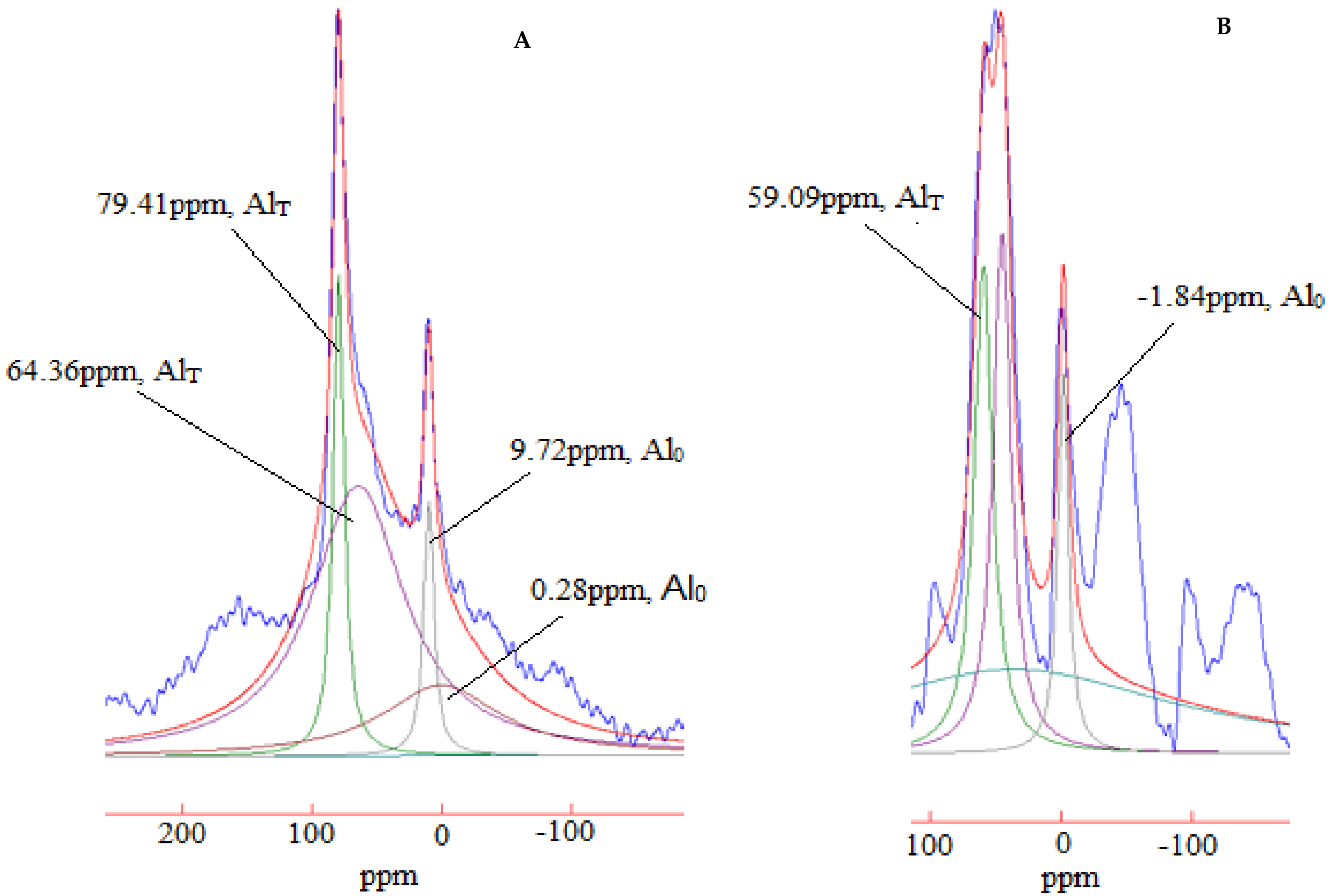

molecules

MDPI

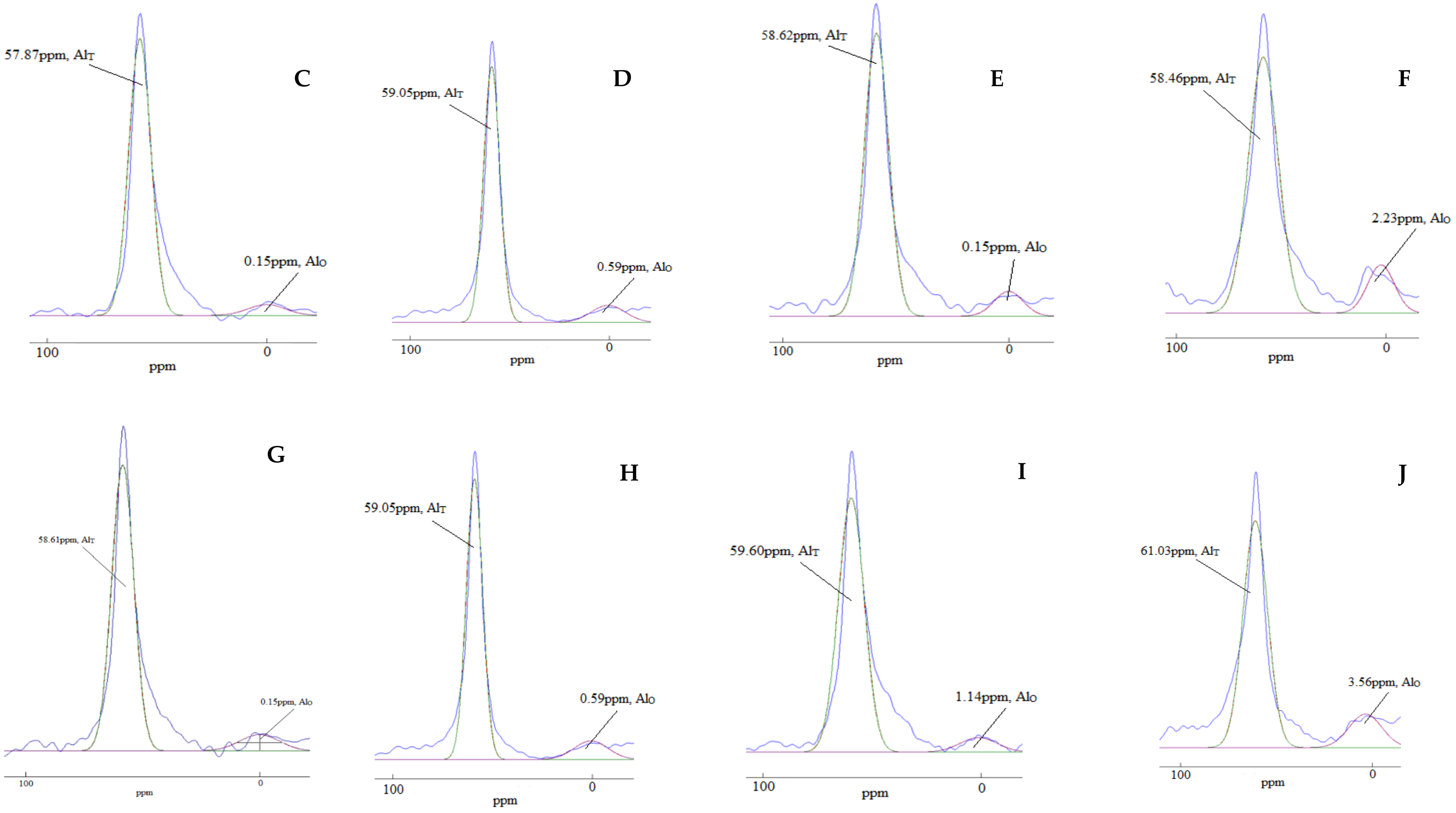

Figure S5. ^{27}Al MAS-NMR spectra for: (**A**) anhydrous OPC; (**B**) anhydrous FA; (**C**) HN T80-2; (**D**) HN T80-28; (**E**) HN T25-2; (**F**) HN T25-28; (**G**) H-WG T80-2; (**H**) H-WG T80-28; (**I**) H-WG T25-2; (**J**) H-WG T25-28
